# TAME 2.0: expanding and improving online data science training for environmental health research

**DOI:** 10.3389/ftox.2025.1535098

**Published:** 2025-02-12

**Authors:** Alexis Payton, Elise Hickman, Jessie Chappel, Kyle Roell, Lauren E. Koval, Lauren A. Eaves, Chloe K. Chou, Allison Spring, Sarah L. Miller, Oyemwenosa N. Avenbuan, Rebecca Boyles, Paul Kruse, Cynthia V. Rider, Grace Patlewicz, Caroline Ring, Cavin Ward-Caviness, David M. Reif, Ilona Jaspers, Rebecca C. Fry, Julia E. Rager

**Affiliations:** ^1^ Department of Environmental Sciences and Engineering, Gillings School of Global Public Health, The University of North Carolina at Chapel Hill, Chapel Hill, NC, United States; ^2^ Center for Environmental Medicine, Asthma and Lung Biology, School of Medicine, University of North Carolina, Chapel Hill, NC, United States; ^3^ The Institute for Environmental Health Solutions, Gillings School of Global Public Health, The University of North Carolina at Chapel Hill, Chapel Hill, NC, United States; ^4^ Curriculum in Toxicology and Environmental Medicine, School of Medicine, University of North Carolina, Chapel Hill, NC, United States; ^5^ Renaissance Computing Institute, The University of North Carolina at Chapel Hill, Chapel Hill, NC, United States; ^6^ School of Data Science and Society, University of North Carolina, Chapel Hill, NC, United States; ^7^ Center for Computational Toxicology and Exposure, US Environmental Protection Agency, Durham, NC, United States; ^8^ Division of Translational Toxicology, National Institute of Environmental Health Sciences, Durham, NC, United States; ^9^ Center for Public Health and Environmental Assessment, US Environmental Protection Agency, Chapel Hill, NC, United States; ^10^ Department of Pediatrics, School of Medicine, University of North Carolina, Chapel Hill, NC, United States

**Keywords:** coding, computational toxicology, data science, data visualizations, exposure science, health research, machine learning, training

## Abstract

**Introduction:**

Data science training has the potential to propel environmental health research efforts into territories that remain untapped and holds immense promise to change our understanding of human health and the environment. Though data science training resources are expanding, they are still limited in terms of public accessibility, user friendliness, breadth of content, tangibility through real-world examples, and applicability to the field of environmental health science.

**Methods:**

To fill this gap, we developed an environmental health data science training resource, the inTelligence And Machine lEarning (TAME) Toolkit, version 2.0 (TAME 2.0).

**Results:**

TAME 2.0 is a publicly available website that includes training modules organized into seven chapters. Training topics were prioritized based upon ongoing engagement with trainees, professional colleague feedback, and emerging topics in the field of environmental health research (e.g., artificial intelligence and machine learning). TAME 2.0 is a significant expansion upon the original TAME training resource pilot. TAME 2.0 specifically includes training organized into the following chapters: (1) Data management to enable scientific collaborations; (2) Coding in R; (3) Basics of data analysis and visualizations; (4) Converting wet lab data into dry lab analyses; (5) Machine learning; (6) Applications in toxicology and exposure science; and (7) Environmental health database mining. Also new to TAME 2.0 are “Test Your Knowledge” activities at the end of each training module, in which participants are asked additional module-specific questions about the example datasets and apply skills introduced in the module to answer them. TAME 2.0 effectiveness was evaluated via participant surveys during graduate-level workshops and coursework, as well as undergraduate-level summer research training events, and suggested edits were incorporated while overall metrics of effectiveness were quantified.

**Discussion:**

Collectively, TAME 2.0 now serves as a valuable resource to address the growing demand of increased data science training in environmental health research. TAME 2.0 is publicly available at: https://uncsrp.github.io/TAME2/.

## 1 Introduction

Data science is a rapidly expanding, interdisciplinary field that integrates methodologies across statistics, computer science, programming, and subject matter expertise to organize, process, and analyze data to build knowledge or solve a problem (NLM, 2024). Data science is now a pillar of environmental health sciences, supporting the successful analysis and integration across an exploding availability of “Big Data,” allowing for integration and interpretation across clinical, epidemiological, social, toxicological, and chemical based evaluations (Choirat et al., 2019; Payton et al., 2023). Only through the effective handling of Big Data and integration across diverse data types will environmental health research better identify environmental drivers of human disease and formulate solutions to alleviate the global burden of disease attributable to environmental exposures (Choirat et al., 2019; Shaffer et al., 2019). Within its current definition provided through the National Institutes of Health (NIH) National Library of Medicine, data science as a field is also highlighted to include the dissemination of data-produced findings through “storytelling, visualization, and other means of communication” (NLM, 2024). Therefore, it is now of increasing importance to train environmental health scientists on data science principles to ensure effective data management, analysis, interpretation, and dissemination, while supporting the underpinnings of fundamental science and research.

Data science training resources are now in increasing demand worldwide. In the field of environmental health, some example agency-level data science training priorities have been outlined by groups including, but not limited to, the NIH Office of Data Science Strategy (NIH, 2024), United States Department of Defense (Hartung et al., 2022), United States Environmental Protection Agency (U.S. EPA) Center for Computational Toxicology and Exposure (Friedman, 2024), and the European Chemicals Agency (European Chemicals Agency, 2016). There is also a growing field of data science pedagogy (Mike et al., 2023; Msweli et al., 2023a; Msweli et al., 2023b), which has highlighted the need for integrating traditional statistics and mathematical training with interpretation of real-world datasets in data science courses (Hicks and Irizarry, 2018; Mike et al., 2023). Furthermore, authors of this article experience first-hand the growing demand for data science training, spanning undergraduates, graduates, post-doctorates, and staff contributing to environmental health research studies.

**FIGURE 1:**
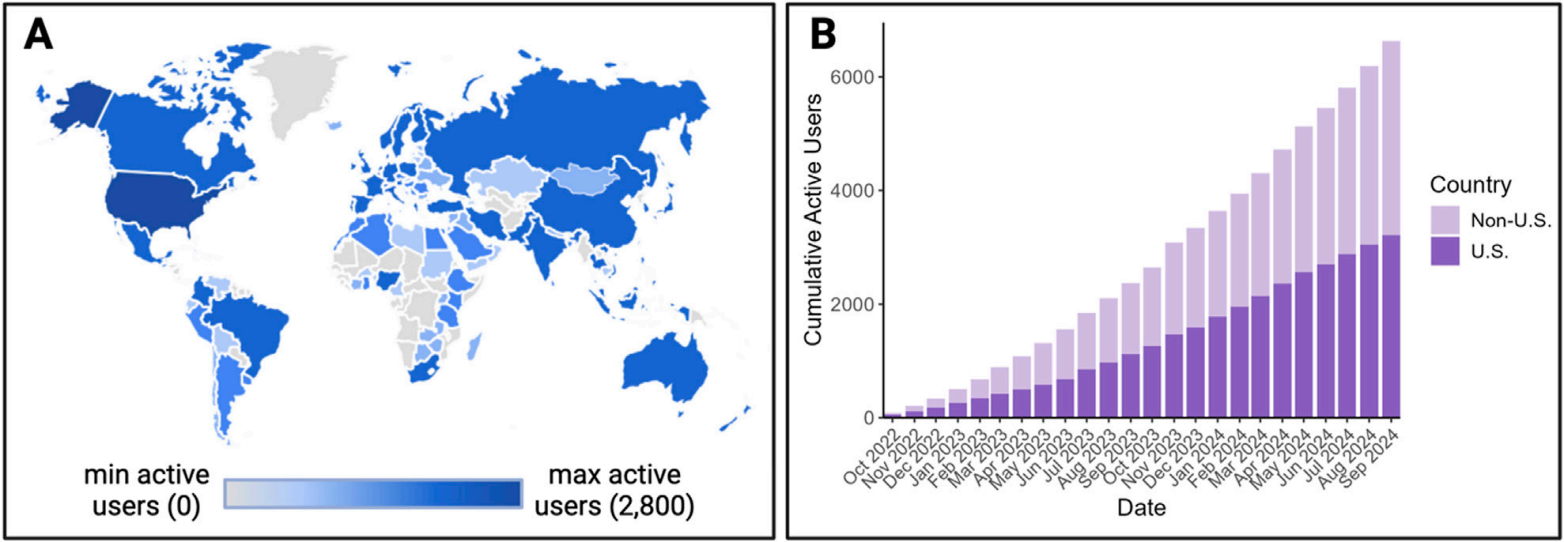
Global usage of the TAME 1.0 website since its launch in 2022. Data are illustrated as **(A)** a worldwide map of users, defined as the number of unique people who have engaged with the website between its 2022 launch up until September 2024. This image was generated through Google Analytics output and a scale was added to enhance interpretability in BioRender Software. Usage is further displayed in **(B)** as the cumulative site views from September 2022 – September 2024 using data aggregated from Google Analytics and plotted in R Software.

To address the growing need for data science training in environmental health, we originally launched the inTelligence And Machine lEarning (TAME) Toolkit, version 1.0 in 2022. The TAME Toolkit is an online training resource that was developed with the goal of promoting trainee-driven data generation, management, and analysis methods to “TAME” data in environmental health research. This publicly available resource was organized through applications-based training modules arranged through a GitHub Bookdown website (Roell et al., 2022b) with underlying script made publicly available (UNCSRP, 2022). Modules were organized across three chapters, the first of which focused on introductory data science information. The second chapter focused on methods to incorporate chemical-biological analyses and predictive modeling efforts into toxicology and health research. The third chapter included examples of environmental health database mining approaches and data integration. This initial launch of the TAME Toolkit was accompanied by a parent publication (Roell et al., 2022a) and has since attracted viewership worldwide as recorded through continuous Google site analytics (Google, 2024) (Figure 1). TAME 1.0 was an important starting point to address the critical need for improved data science training resources, and authors have always acknowledged the need for this resource to be expanded upon and updated as the field continues to rapidly progress.

## 2 Materials and methods

### 2.1 Process of prioritizing new training and content expansions

TAME 2.0 content was designed to address data analysis training needs across a wide range of skill levels, starting with basics of data organization and scripting in R up to and including more complex analyses and applications in environmental health science, such as machine learning (ML) and mixtures modeling. Content was prioritized based on observations of the research team and worldwide agency-level data science training priorities. Research team observations included day-to-day interactions with trainees, colleague input, the peer review process, and current events such as the rise in popularity of artificial intelligence (AI) and ML, including natural language processing tools such as ChatGPT. Additional priorities across students, research teams, and agencies included the following: increased training on topics such as statistics and mathematics, improving data visualizations, transitioning between laboratory-based research to computational research, effective statistical method selection, and developing standards for data analysis approaches.

### 2.2 Generation of TAME 2.0 training materials

To describe the methods and underlying software used to generate TAME 2.0, training content was drafted in R Markdown using a codified system, and a bespoke style guide was implemented to improve content ordering and visualization consistencies across modules (see Supplementary Slides). Although each module was allowed flexibility, all modules started with an introduction to the module’s topic to provide context and, when needed, domain-specific introductory materials and terminology. Then, a section was dedicated to informing the audience about the content to be explored in the module, which included background on example dataset(s) used throughout the scripted activities. Most modules also included a list of environmental health questions that were answered throughout the module using code, with the goal of engaging audience participation as viewers. Each environmental health question was answered, and supporting text was provided to assist the reader in connecting the concept with its execution within the code and associated annotation. Graphical overviews were provided when needed and were created using Biorender.com. All modules were rendered via Bookdown and are publicly available online and through the University of North Carolina at Chapel Hill (UNC) SRP GitHub repository (UNCSRP, 2024a). Lastly, modules were wrapped up by providing additional resources for further reading and ‘Test Your Knowledge’ (TYK) questions. These TYK questions provide trainees with additional example datasets to test what they were able to learn from the module in an additional environmental health research application. Input datasets are available in a folder within the TAME 2.0 GitHub repository (UNCSRP, 2024c), and solutions to these TYK questions, along with code showing how to answer each question, are provided as a separate GitHub repository (UNCSRP, 2024b). These solutions, including the dataset(s) and code, can be downloaded from the GitHub repository if trainees prefer to run them locally on their own computers.

### 2.3 Testing and disseminating TAME 2.0 training materials

Many of the training modules were used as the basis for course materials for a graduate-level course at UNC titled “ENVR730: Computational Toxicology and Exposure Science.” Draft materials were presented as the content for lectures, and TYK activities were used as homework. The course survey was organized such that all classroom participants (n = 25) filled out questionnaires at the start of the semester, and at the end of the semester. Questions were designed to understand students’ level of familiarity with toxicity and exposure databases, ability to apply general data analysis techniques, and ability to describe or apply analysis techniques specific to environmental health questions (Supplemental Table S1). Responses were on a Likert scale, with lower responses indicating less familiarity or ability, and higher responses indicating higher familiarity or ability. Administration and dissemination of findings from this course survey were reviewed by the UNC IRB and deemed non-human subject research due to subject deidentification protocols (IRB 24-1197). Survey results and training efficacy statistics/figures in this manuscript focus on the course evaluation. Responses were compared pre vs. post course completion using paired Wilcox tests and visualized using box plots and stacked bar charts in R. Additional dissemination of training efforts (summarized in Supplemental Table S2) were coordinated through the following: (1) in-person data science training workshops (two workshops presented to a total of 60 participants), such as our PRogramming for Environmental HEalth And Toxicology (PREHEAT) Retreat (October 20–21, 2022 at UNC); (2) longer research training programs (two programs, presented to a total of 25 participants), such as a summer-long Equity and Environmental Justice Program (QUEST) internship, a paid summer experience for undergraduate students interested in environmental justice-based research (yearly at UNC); (3) conference dissemination (four conferences of >500 attendees each), where we often describe how data analysis methods implemented in the discussed research are described through TAME training modules; and (4) promotion of the TAME Toolkit in dedicated presentations nation-wide, including presentations at the NIH, U.S. EPA, and several academic institutions (three presentations, presented to a total of 90 participants). Survey results and training efficacy statistics/figures in this manuscript focus on the course evaluation since it underwent IRB approval, though informal findings from workshops and other mechanisms of dissemination are provided via narrative text and were considered throughout development and refinement of TAME 2.0.

## 3 Results

### 3.1 Improved training content

**FIGURE 2:**
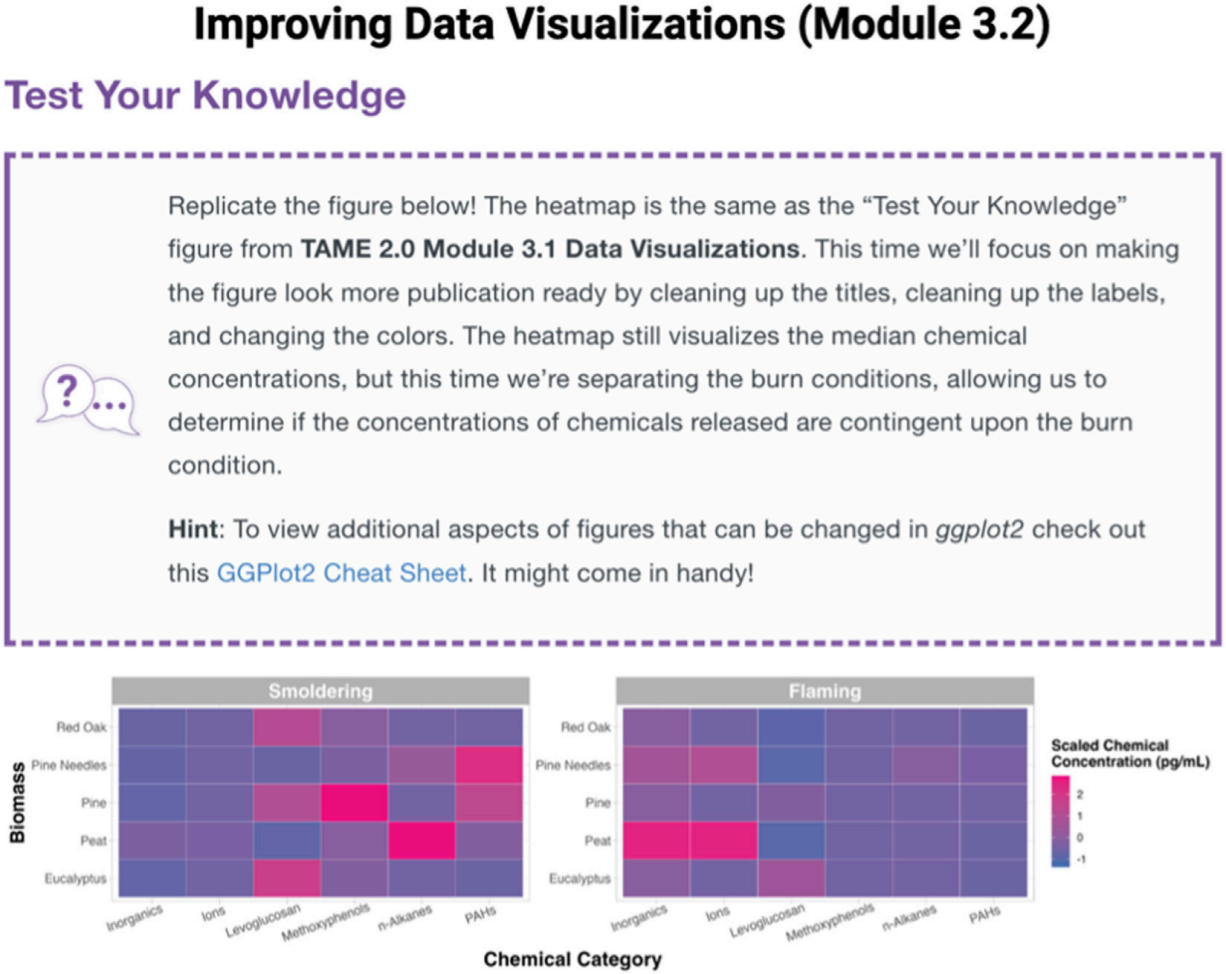
Example Test Your Knowledge box located at the end of Module 3.2, “Improving Data Visualizations.” These “take-home” assignments were designed to further engage participants and test their skillset related to the topics discussed within each training module. Test Your Knowledge boxes represent a new addition to TAME 2.0, developed to address requests for more hands-on assignments.

Training content improvements and expansions implemented within TAME 2.0 were prioritized based upon the review of information obtained from students/trainees, members of our research team, and agency documents, as described in the Methods. Based upon this review and brainstorming between our team members, we prioritized eight topics to add and/or expand within TAME 2.0. These topics are listed in Table 1, alongside specific steps we coordinated to address these prioritized edits to TAME. As an example, trainees expressed interest in obtaining more hands-on training exercises. To address this goal, we added “Test Your Knowledge” boxes at the end of each training module to provide participants more opportunities to apply concepts that they learned throughout each training lesson towards a new research question and/or concept (Figure 2).

**TABLE 1:** Data science training topics that were prioritized to either be added or expanded upon within TAME 2.0. The specific steps that were carried out to address each of these priorities are listed as well as their corresponding locations within TAME 2.0.

New and/or expanded training content priorities	Steps to address this priority implemented in TAME 2.0	Location(s) of new training materials in TAME 2.0
Expanding data science training content, in general	Expanded content was developed, including the generation of entirely new modules to enhance data analysis training	Throughout all Chapters
Current standards and “best practices” for data management and analysis	Presented resources discussing current data analysis standards, including a new module on script version tracking and sharing. Training on FAIR guidance principles was also expanded	A focus of Modules 1.1 and 1.3, with additional training throughout Chapters 1 and 2
Hands-on training exercises	Incorporated new “Test Your Knowledge” boxes that ask participants questions about the training topic and/or analysis of a dataset using the materials provided in the module	At the end of each individual training module (when applicable)
Improving ways to visualize data	Provided new guidance on creating “publication worthy” figures and tables throughout scripted activities	A focus of Module 3.2, with additional training throughout Chapter 4 and Modules 6.1 and 6.2
Increasing narrative and didactic content	Included more schematics and figure overviews to explain and narrate data processing steps and analysis concepts more clearly	Each module’s introduction and throughout each module
Training on “hot topics” in data science (e.g., AI/ML)	1) Introduced the history and context of artificial intelligence, machine learning, and predictive modeling used in environmental health and developed illustrative applications of these techniques using real data 2) Expanded the number of machine learning modules and topics covered, such that machine learning modules now represent an entire chapter dedicated to this topic	Chapter 5
Training on statistical method selection	Discussed statistical tests for normality, variance, multiple group comparisons, and data distributions. Also incorporated a flow chart to aid in deciding which statistical test to run depending on experimental design factors	Modules 3.3–3.4 and 4.4–4.6
Transitioning wet bench data to computational (“dry lab”) analyses	Demonstrated how computational methods can be used to clean, analyze, and report data generated through wet bench experimentation for improved reproducibility and transparency	Chapter 4

### 3.2 Overview of TAME 2.0

**FIGURE 3:**
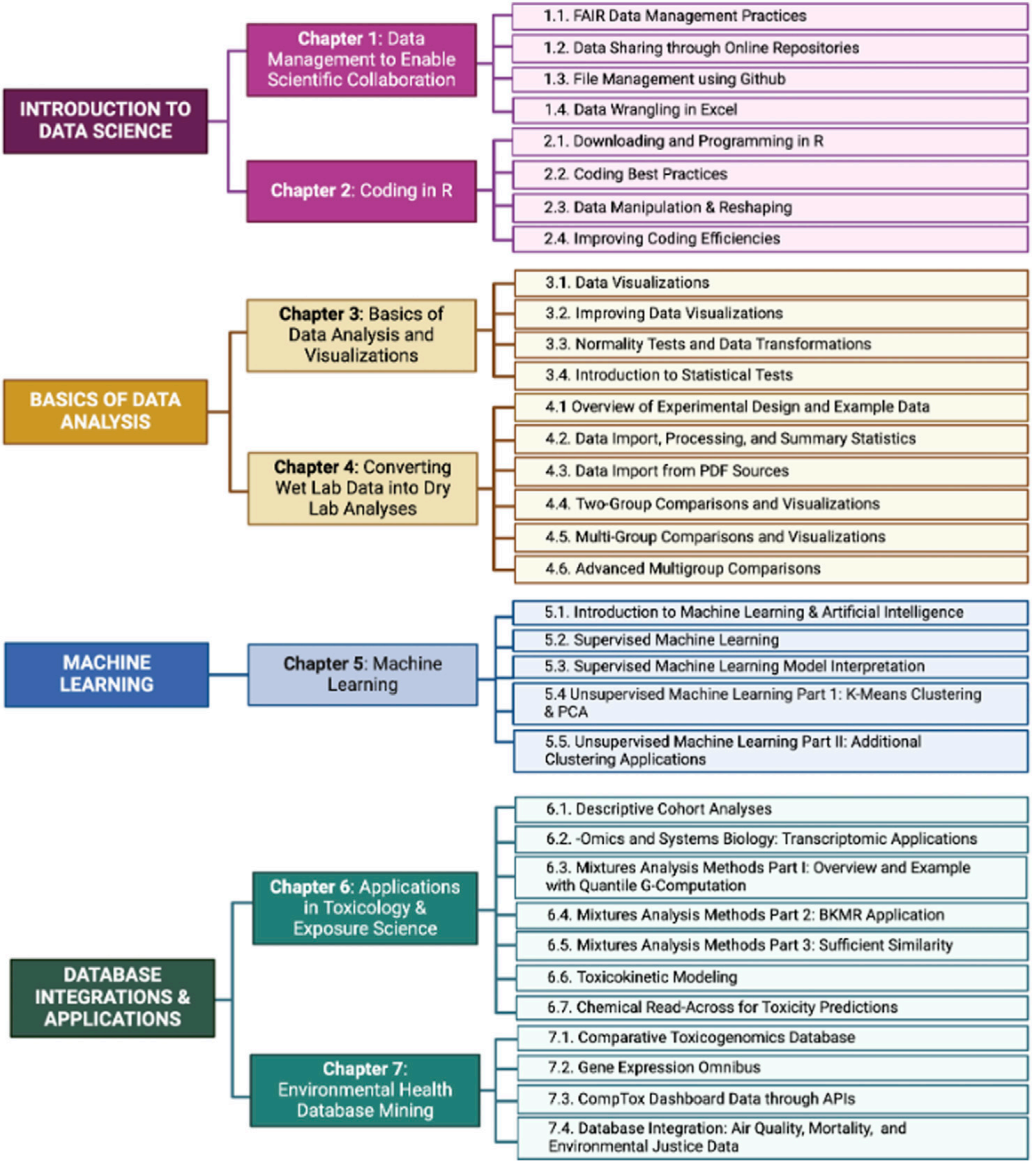
Overview of the current main themes, chapters, and individual modules contained in TAME 2.0. Main themes are noted on the left side of the flow diagram, chapters in the middle, and individual modules are listed on the right.

TAME 2.0 is organized across seven chapters, which address broad topics in data science training–Introduction to Data Science, Basics of Data Analysis, Machine Learning, and Database Integrations and Applications (Figure 3). Within each chapter, individual modules encompass specific topics related to that chapter’s theme. Modules are generally designed to stand alone, though some modules may require working knowledge of content presented in previous chapters/modules, acquired through review of earlier TAME chapters and/or from other prior training experience. For example, a user who has not coded previously could begin TAME-based training in Chapter 1, while a user who has familiarity with R programming and basic analyses could begin training within later modules. Module content and recommendations are based on the authors’ expertise, and throughout modules, it is emphasized to consider best practices and approaches in the participant’s research group and/or field of study. A high-level description of each chapter is provided below. Note that all references for specific packages and example datasets are clearly reported within the TAME 2.0 website.

#### 3.2.1 Chapter 1: data management to enable scientific collaboration

**FIGURE 4:**
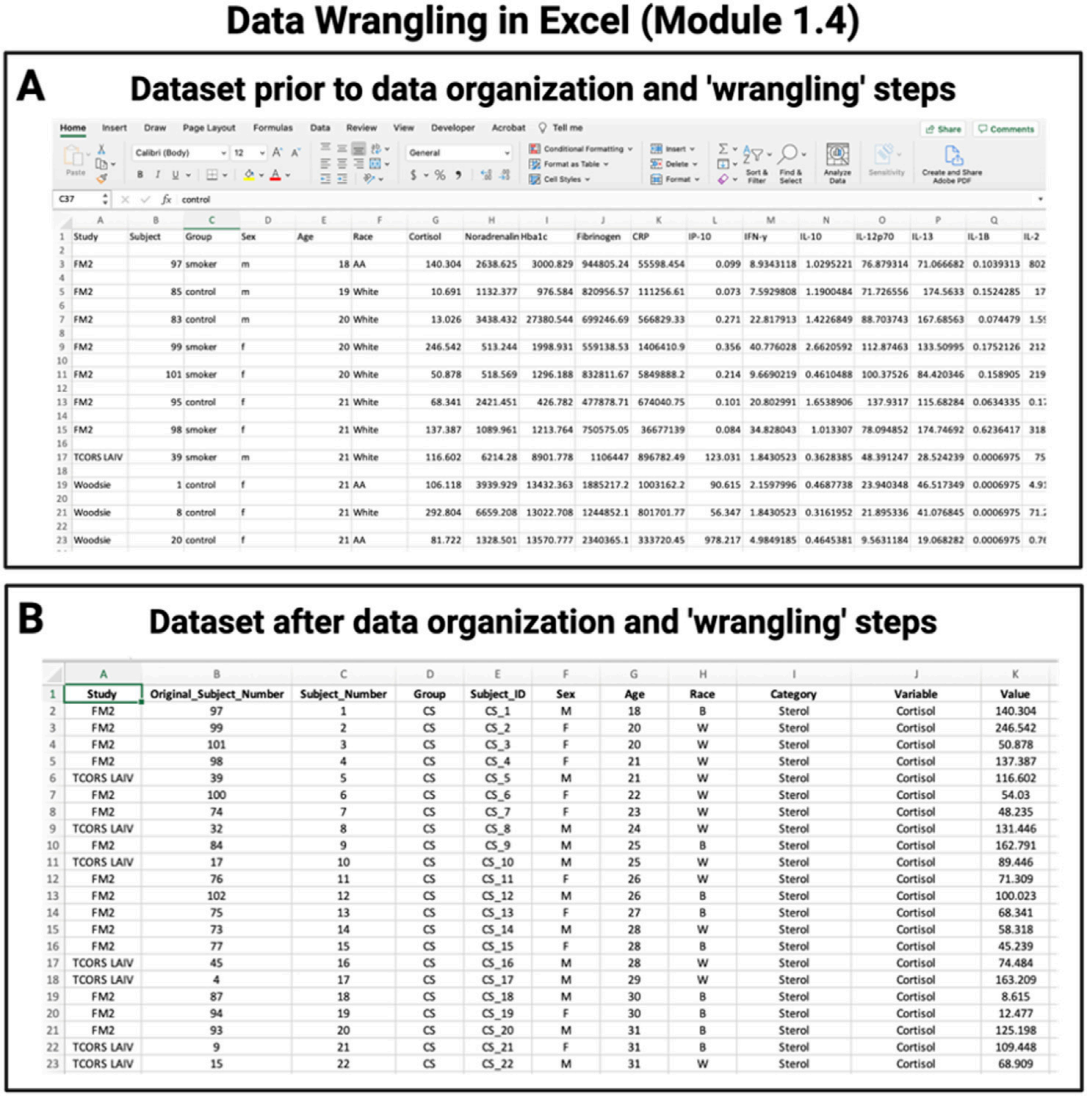
Example training activity in Chapter 1, highlighting Module 1.4 “Data Wrangling in Excel”. Participants are provided guidance on approaches to organize data for example, converting raw, unorganized data **(A)** into organized data ready for analysis **(B)** to enhance data sharing, interoperability, and amenability towards downstream analyses.

The goal of Chapter 1 is to introduce practices and platforms that enable data sharing for the scientific community. The first module (1.1) describes principles that constitute proper data management based on Findability, Accessibility, Interoperability, and Reusability (FAIR) guidance principles (Wilkinson et al., 2016). Part of what makes data FAIR includes publishing datasets in publicly accessible online repositories, which are discussed in Module 1.2. These principles are applicable to code as well and Module 1.3 demonstrates file management with GitHub, the most commonly used resource for depositing, sharing, and collaborating on code. The chapter concludes with Module 1.4, which provides examples for wrangling data in Microsoft Excel to make data files more amenable to performing downstream analyses, especially when using programming languages (Figure 4). This chapter collectively serves as a resource for best practices when sharing, publishing, and formatting data for easier dissemination and analysis in environmental health research.

#### 3.2.2 Chapter 2: coding in R

**FIGURE 5:**
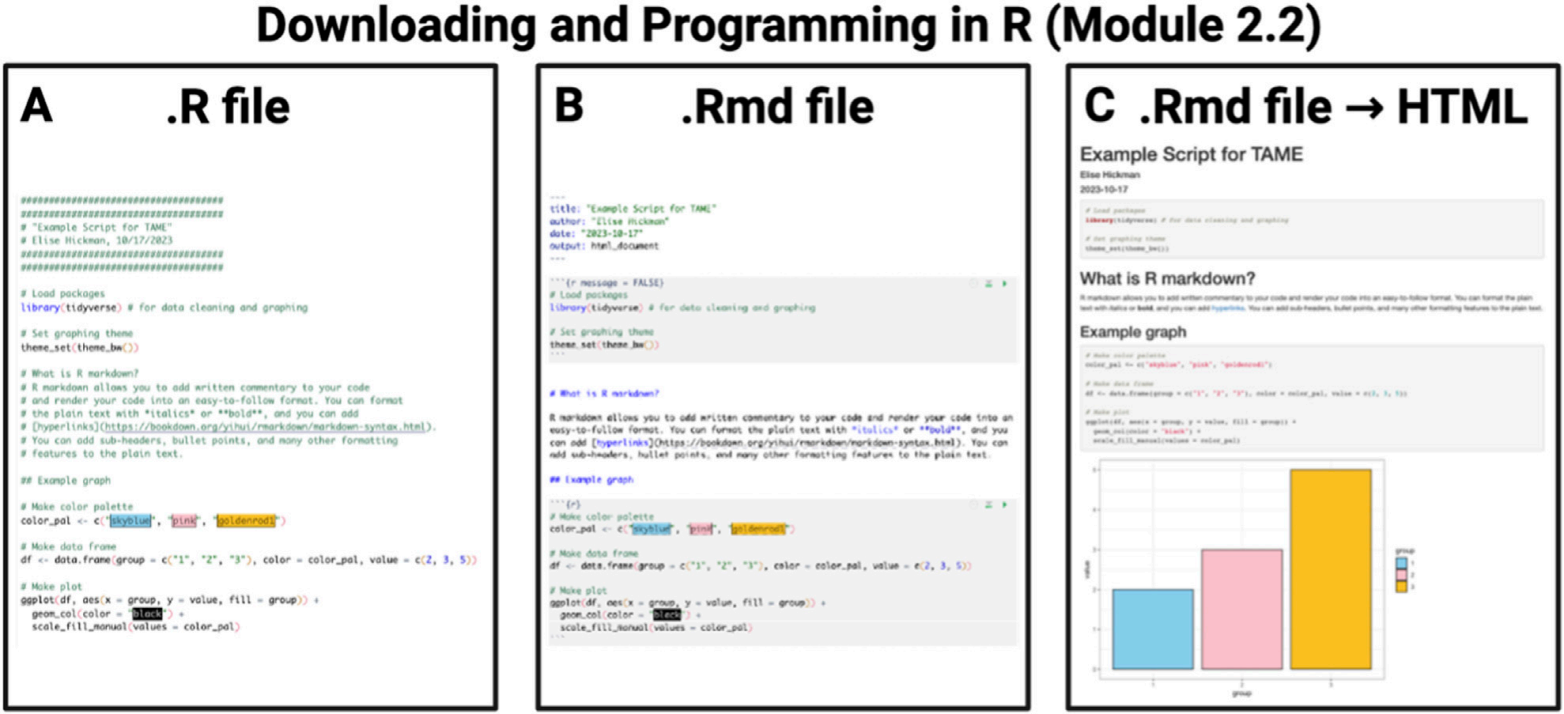
Examples of R files. RStudio, the software commonly used to run R, can execute script using a few file types including **(A)**. R, **(B)** R Markdown (.Rmd), and **(C)**. html, which are reviewed in TAME 2.0 Module 2.2.

Chapter 2 introduces the programming language R, which is commonly used for analyzing data across many applications, including environmental health research, and is used in all of the following modules and chapters. Module 2.1 serves as a step-by-step guide to walk readers through the basics of downloading R and RStudio, how to use the RStudio interface, and basic coding terminology and concepts. This introduction is followed by Module 2.2, which describes example “best practices” for coding. This module first describes differences between script file types (e.g.,. R script versus R markdown. Rmd) (Figure 5) and then reviews script naming, annotation, organization, and coding styles. Next, the chapter shifts its focus to walk through reshaping and manipulating data as needed for succeeding analyses and visualizations in Module 2.3. The final module, 2.4, introduces approaches to improve coding efficiencies through more advanced coding skills, such as loops and functions. In summary, this chapter helps participants become more familiar with coding in R, the RStudio interface, and ways to organize data and script files for increased interpretability, data tracking, and ease of downstream coding exercises.

#### 3.2.3 Chapter 3: basics of data analysis and visualization

**FIGURE 6:**
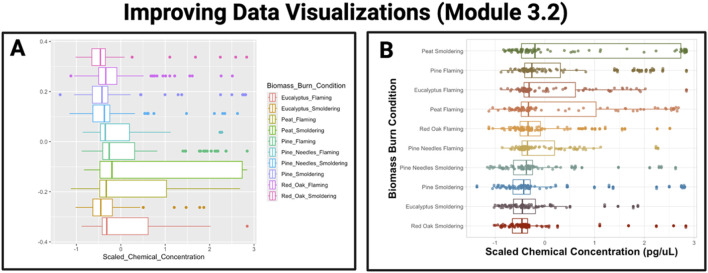
Example training activity in Chapter 3 demonstrates an approach to improve data visualizations. The example graphic provided on the left **(A)** serves as a demonstration of a boxplot that results from running default parameters within the *ggplot2* package, using data from a recently published dataset (Rager et al., 2021). The example graphic provided on the right **(B)** serves as an example improvement of the boxplot visualization, where *ggplot2* parameters were modified to increase font size, use a custom color scheme, relabel axes, and order boxes from highest to lowest scaled median chemical concentration.

The purpose of this chapter is to discuss introductory-level data analyses and visualizations and to introduce techniques to improve the organization and presentation of tables and figures, thus making them “publication-ready.” In the first module (3.1), examples are provided to guide readers through various types of basic plots that can be constructed using the R package, *ggplot2*, including density plots, boxplots, heatmaps, and correlation plots to explore distributions of an example dataset. Next, Module 3.2 highlights methods to communicate scientific findings in more succinct and visually appealing ways as demonstrated in Figure 6. The second half of the chapter transitions into initial quantitative assessment of data, introducing the concept of normality and data transformations (Module 3.3) before diving into basic statistics including t-tests, analysis of variance (ANOVA), regression modeling, and categorical tests (Module 3.4). Importantly, this chapter also demonstrates examples of appropriate figure legends and how to incorporate results from data exploration and initial workflow steps such as assessing normality into a manuscript suitable for peer review. This chapter overall represents an introductory-level approach to critical elements of qualitative and quantitative evaluation of a dataset’s distribution, with the ultimate goal of preparing scientists to coordinate baseline data analyses and visualizations.

#### 3.2.4 Chapter 4: converting wet lab data into dry lab analyses

**FIGURE 7:**
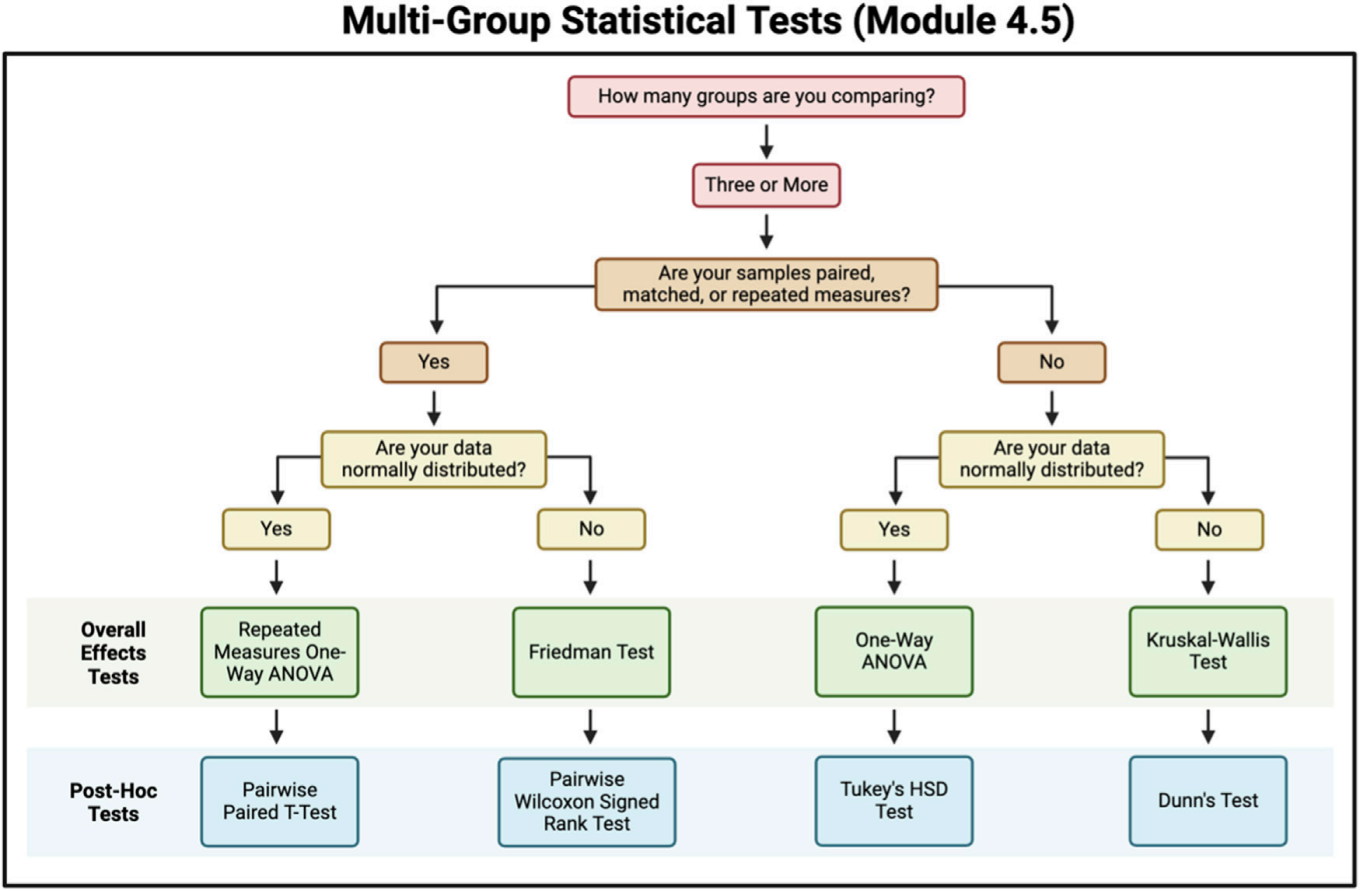
Flowchart for selecting a multi-group statistical test. This figure contains questions for criteria to be met to help select a multi-group statistical test to use.

This chapter is specifically intended for wet lab-focused scientists (or their computational collaborators) who need to wrangle data and perform analyses. These steps are often completed using subscription or license-based applications, including but not limited to Prism, SAS, and Spectronaut, which are not always accessible due to financial barriers and/or lack workflow transparency. To address this, the modules in Chapter 4 provide examples demonstrating how to perform similar analyses using R. Module 4.1 provides an overview of experimental design, including replicates, and it introduces the example data that will be used for the subsequent modules in Chapter 4. Modules 4.2 and 4.3 demonstrate reading in various file types into R that are common outputs of wet lab experiments, handling missing data examples, and averaging replicates. Notably, Module 4.3 specifically discusses importing PDF files, which can be challenging in R. Modules 4.5–4.7 provide in-depth descriptions of statistical tests and guidance surrounding how to choose the correct test for your experimental design and hypothesis (Figure 7). The last three modules not only highlight two group and multi-group comparisons and visualizations but also, similar to the modules in Chapter 3, provide additional content demonstrating how to present the results in publication-ready tables and figures. Overall, Chapter 4 is designed for wet bench scientists aiming to transition experimental data to analysis in R and provides examples of common analysis steps, such as reading in data, cleaning data, and implementing commonly used statistical tests.

#### 3.2.5 Chapter 5: machine learning

**FIGURE 8:**
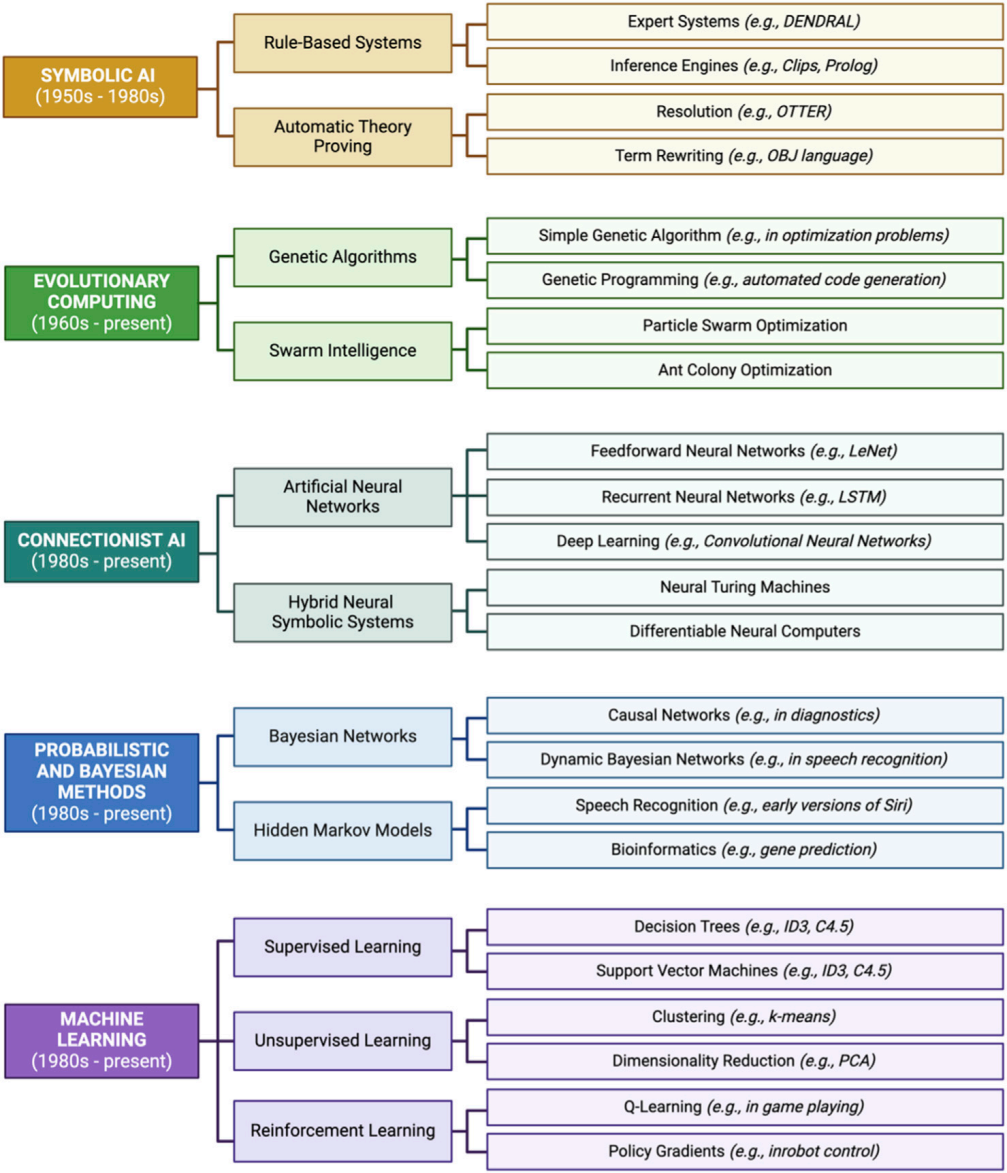
Historical timeline of events and methods leading up to modern-day applications of artificial intelligence/machine learning (AI/ML) in environmental health research. Listed here are relevant examples of events starting in the 1950s and ending with present AI/ML methods.

Chapter 5 lays the groundwork for applications of ML and AI algorithms and reviews example approaches commonly implemented in the field of environmental health science. The first module (5.1) reviews the history of ML, including events and methods that have led up to current applications of AI/ML in environmental health research (Figure 8). Module 5.1 also discusses the many buzzwords strongly associated with AI/ML, including a breakdown of AI/ML taxonomy and further describes the use of AI/ML in research aimed at predicting chemical hazards and associated health risks of environmental exposures. Next, Module 5.2 reviews supervised ML and important concepts necessary to build a successful model, including cross validation, types of algorithms, and confusion matrix performance metrics. To emphasize the interpretability of supervised ML models, Module 5.3 provides explanations of variable importance and decision boundary figures. The next two modules (5.4 and 5.5) highlight applications for unsupervised ML, including examples that demonstrate how to identify patterns in data through clustering (e.g., *k*-means) and data reduction (e.g., principal component analysis). In summary, Chapter 5 highlights environmental health-relevant applications for ML and how to improve accessibility of the resulting findings for researchers across diverse scientific backgrounds.

#### 3.2.6 Chapter 6: applications in toxicology and exposure science

**FIGURE 9:**
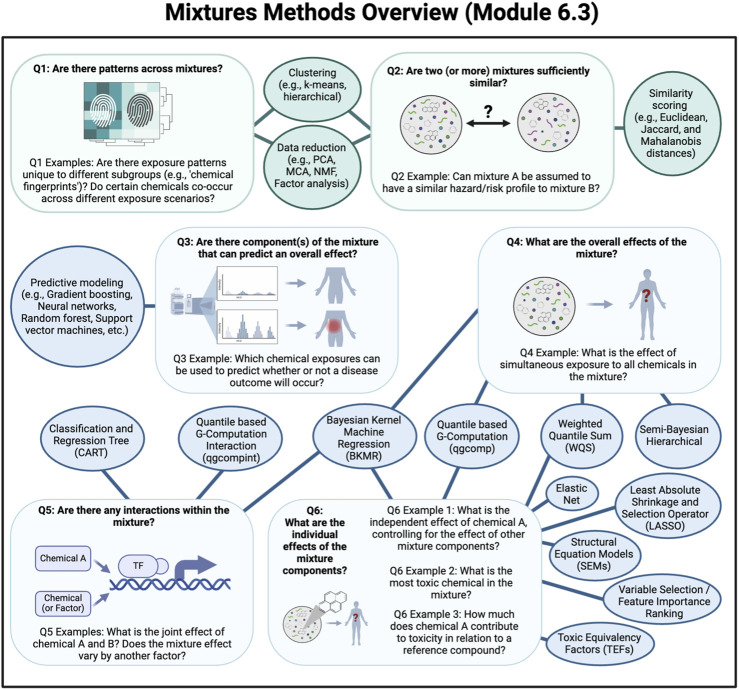
Mixtures analysis methods for specific types of research questions. This infographic depicts analysis approaches that can be used when assessing the impact of chemical mixtures on health outcomes.

Chapter 6 provides examples of various analysis approaches that can be executed through R script to analyze epidemiology, toxicology, and exposure science applications, including mixtures assessments. The chapter starts with a module that demonstrates data cleaning, filtering, and summary steps for descriptive human cohort analyses (6.1), followed by a module that walks through a transcriptomic analysis (6.2). Modules 6.3, 6.4, and 6.5 are focused on mixtures analysis methods and provide examples of quantile g-computation, Bayesian kernel machine regression, and sufficient similarity, respectively (Figure 9). Then, module content transitions to computational modeling approaches; specifically, toxicokinetic modeling with the *httk* R package (6.6) and chemical read-across with the *fingerprint* and *rcdk* R packages (6.7). Collectively, this chapter represents many ways in which coding strategies can be used to answer important questions in environmental health using advanced approaches and provides essential background and steps needed to execute these analyses.

#### 3.2.7 Chapter 7: environmental health database mining

**FIGURE 10:**
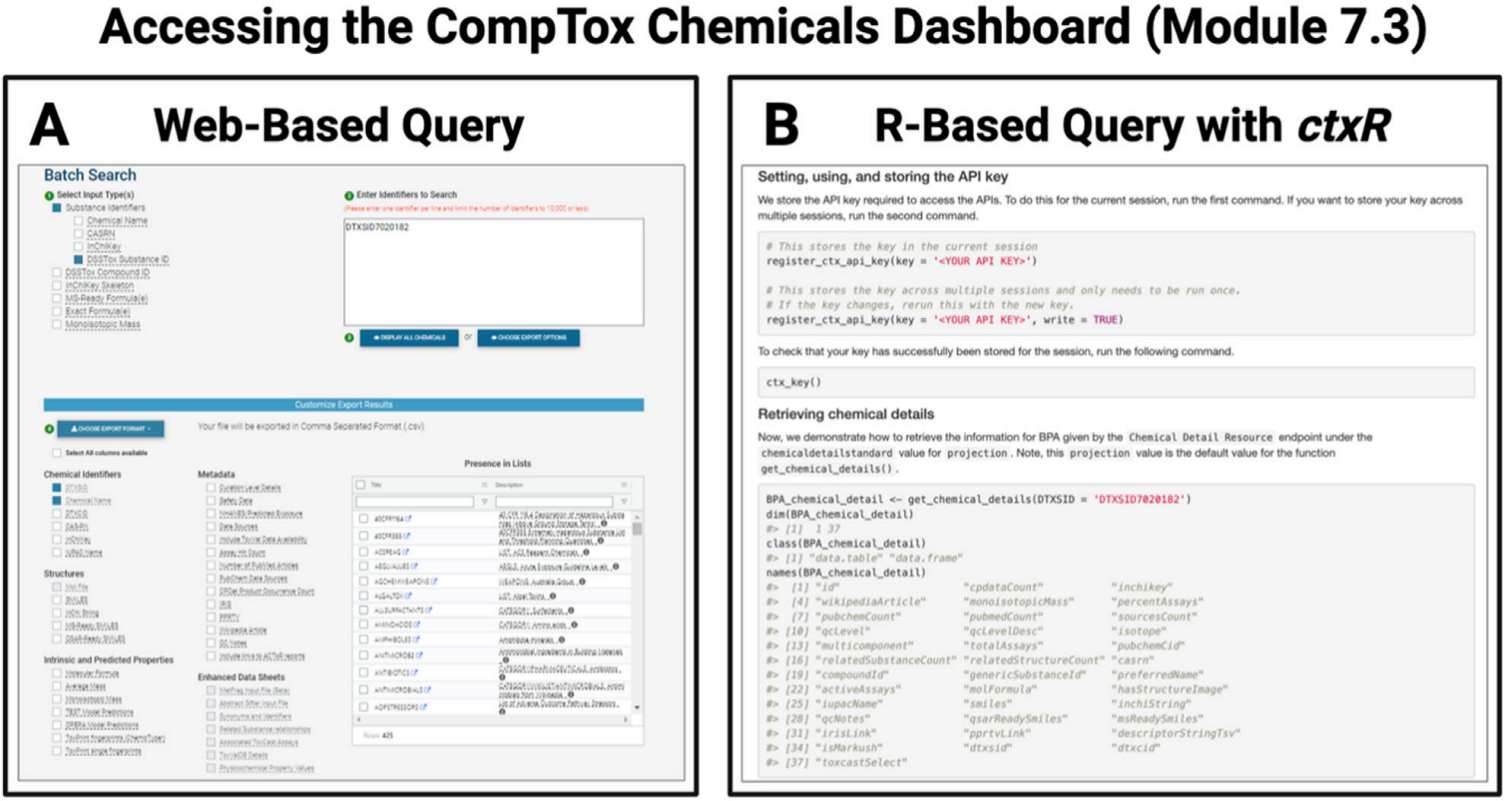
Example of database query options available to more quickly parse large datasets organized through the CompTox Chemicals Dashboard. Chapter 7 focuses on integrating databases into scripted analyses, as exemplified by screenshots presented here from Module 7.3, which demonstrates how to access the CompTox Chemical Dashboard using **(A)** a traditional web-based query and **(B)** the newly available R-based query with the *ctxR* package.

The last chapter highlights methods to integrate data across several major environmental health databases through scripted analyses. In Module 7.1, the Comparative Toxicogenomics Database (CTD) (Davis et al., 2021) is featured, with an example demonstrating how to access CTD data and perform a simple analysis of those data in R. Module 7.2 follows a similar structure and focuses on accessing and analyzing data housed within the Gene Expression Omnibus (GEO). Next, Module 7.3 demonstrates how to access data from the CompTox Chemicals Dashboard (Williams et al., 2017) both through manual web-based query and through the recently developed *ctxR* package, which allows users of the CompTox Chemicals Dashboard to access the dashboard through an Application Programming Interfaces (API) request (Figure 10). This module also demonstrates a variety of environmental health-based analyses that are reliant upon substance chemistry and toxicity that can be conducted once data have been acquired. Chapter 7 culminates with Module 7.4, which provides a scripted example of integrating data from multiple databases U.S. (Author anonymous, 2024; County Health Rankings, 2010; U.S. Environmental Protection Agency AirData, 2025) to assess air quality, mortality, and environmental justice. Altogether, the modules in this chapter provide trainees with knowledge that is needed to begin working with publicly available databases, a skill that is useful across scenarios such as validating experimental findings, integrating findings across groups, and generating new hypotheses.

### 3.3 Testing and disseminating training materials

**FIGURE 11:**
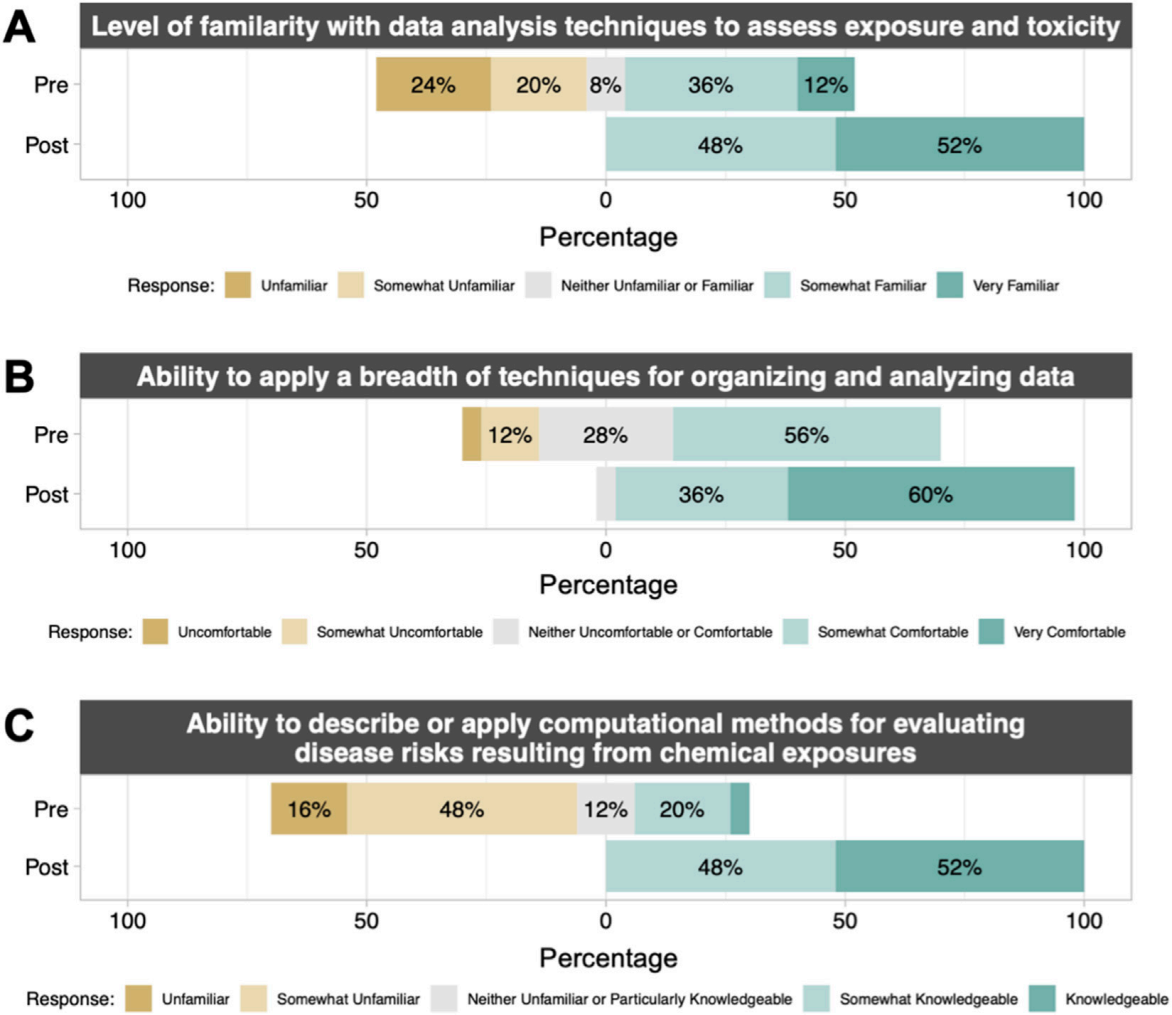
Pre- and post-course student survey results from UNC “ENVR730: Computational Toxicology and Exposure Science.” Students filled out a survey before beginning the course and after completing the course to understand changes in their **(A)** familiarity with and **(B, C)** ability to describe or apply computational methods in toxicology and exposure science. The complete survey included 11 questions; 3 representative questions and results are shown here. For full results, including statistical comparisons pre-vs. post-course, see Supplemental Figures. All comparisons pre vs. post were significant with p < 0.01 by paired Wilcox test. n = 25 students with matched pre/post results.

TAME 2.0 material effectiveness was evaluated both quantitatively and qualitatively through engagement across students, staff scientists and researchers, and principal investigators. To quantitatively assess the efficacy of training, a course survey was specifically administered to a class of graduate-level students (n = 25) enrolled in Dr. Rager’s course in the Fall of 2023, titled “ENVR730: Computational Toxicology and Exposure Science”. Materials used in the course consisted of training materials organized within TAME 2.0, and survey responses were collected pre vs. post course completion. Students reported significantly increased (p < 0.01) scores for all questions on the survey after taking the course (Supplemental Figure S1; Supplemental Table S3). Notably, students reported an increased level of familiarity with data analysis techniques to assess exposure and toxicity, increased comfort with applying a breadth of techniques (including Excel and R script) to analyze and visualize data, and increased knowledge of methods to evaluate chemical exposure associated disease risks and chemical-disease relationships (Figure 11, Supplemental Figures S2–S4). On a qualitative basis, we received feedback through multiple additional mechanisms of TAME 2.0 dissemination, spanning data science training workshops, longer research training programs, conference dissemination, and promotion of TAME 2.0 through dedicated presentations across the nation. Collectively, narrative-level feedback from these efforts tended to highlight the appreciation for such a resource to continue to expand, as trainees continue to communicate their interest in more data science training. Engaged audience members and workshop participants noted that the website was visually pleasing and that the training materials were welcoming towards participants of diverse backgrounds and levels of comfort surrounding coding.

## 4 Discussion

After the successful launch of the first version of the inTelligence And Machine lEarning (TAME) Toolkit in 2022, we sought to continue to promote data analysis competencies needed in environmental health research through expanded training materials that are now presented as an updated version of TAME, namely TAME 2.0. In the spirit of its predecessor — to equip trainees with skills required to “TAME” data — relevant topics for inclusion were identified using undergraduate and graduate feedback in workshops and coursework, as well as input from experts and agencies within the field. Based on these insights, we released TAME 2.0, which includes expanded data science training content, hands-on training exercises, and a focus on communicating and disseminating results of analyses. The effectiveness of these training modules was subsequently demonstrated using surveys administered to graduate students prior to and following the implementation of the training program at UNC. This quantitative approach to evaluating effectiveness supports TAME 2.0 as one of the first educational resources to report educational data regarding its reach and effectiveness in the field of environmental health.

It is notable that data science training resources are currently expanding. For example, the National Institute of Environmental Health Sciences (NIEHS) recently posted a list of current resources for environmental health science data training (NIEHS, 2023), which mentioned our initial TAME resource. The majority of these resources are spread across different organizations and formats and often cover specific data science skills rather than illustrating applications of these skills to examples within environmental health. In contrast, TAME 2.0 is intended to span introductory to advanced applications and integrates didactic content, narrated scripted analyses in environmental health, and independent practice. Thus, materials across the TAME collection continue to provide unique training resources for participants engaged in environmental health research.

While TAME 2.0 gives an illustrative introduction to various data analysis methods, it is not intended to be exhaustive. Where relevant, the toolkit includes links to additional resources for trainees regarding R programming and in-depth explanations of analysis methods. Notably, the goal of TAME is not to prescribe specific analysis methods or styles to environmental health data but rather to demonstrate relevant approaches based on our coding and data analysis experiences. Others within the field may be preferential to other methods and styles, which is acknowledged throughout the modules, and readers are encouraged to consider standard approaches in their line of research or research group. Additionally, TAME 2.0 was scripted in the R coding language because R is an open-source language with relevant packages and compressive documentation, ideal for collaboration, reproducibility, and transparency. Due to these attributes, R has become a popular language for environmental health researchers. In the future, we seek to provide training resources in other coding languages, such as Python, to further increase accessibility of scripted analyses and leverage packages and methods that may be unique to specific coding languages. Lastly, measuring the effectiveness of data analysis training, such as TAME modules, is an ongoing challenge. In the future, assessment of effectiveness of data science training is needed, including both quantitative outcomes such as learner retention, time to competency for a module, and time to completion for a specific application based on engagement with the TAME website and qualitative feedback regarding trainees’ experiences using TAME 2.0.

In conclusion, TAME 2.0 represents a significant expansion on the original TAME resource and enables participants to independently gain skills relevant to environmental health data management and analysis. TAME 2.0 serves as an example of how training materials can be constructed to meet the ever-expanding needs for data science training, paving the way for future efforts, which will improve the capabilities of the research workforce and increase the quality and reproducibility of research studies in environmental health.

## Data Availability

The raw data supporting the conclusions of this article will be made available by the authors, without undue reservation.
