# Supplementary material for: TAME 2.0: expanding and improving online data science training for environmental health research
Source: Front Toxicol. 2025 Feb 12;7:1535098. doi: 10.3389/ftox.2025.1535098 (PMC11860945; doi:10.3389/ftox.2025.1535098)
Supplement: Supplementary file 1 [file Presentation1.pptx]

## Slide 1
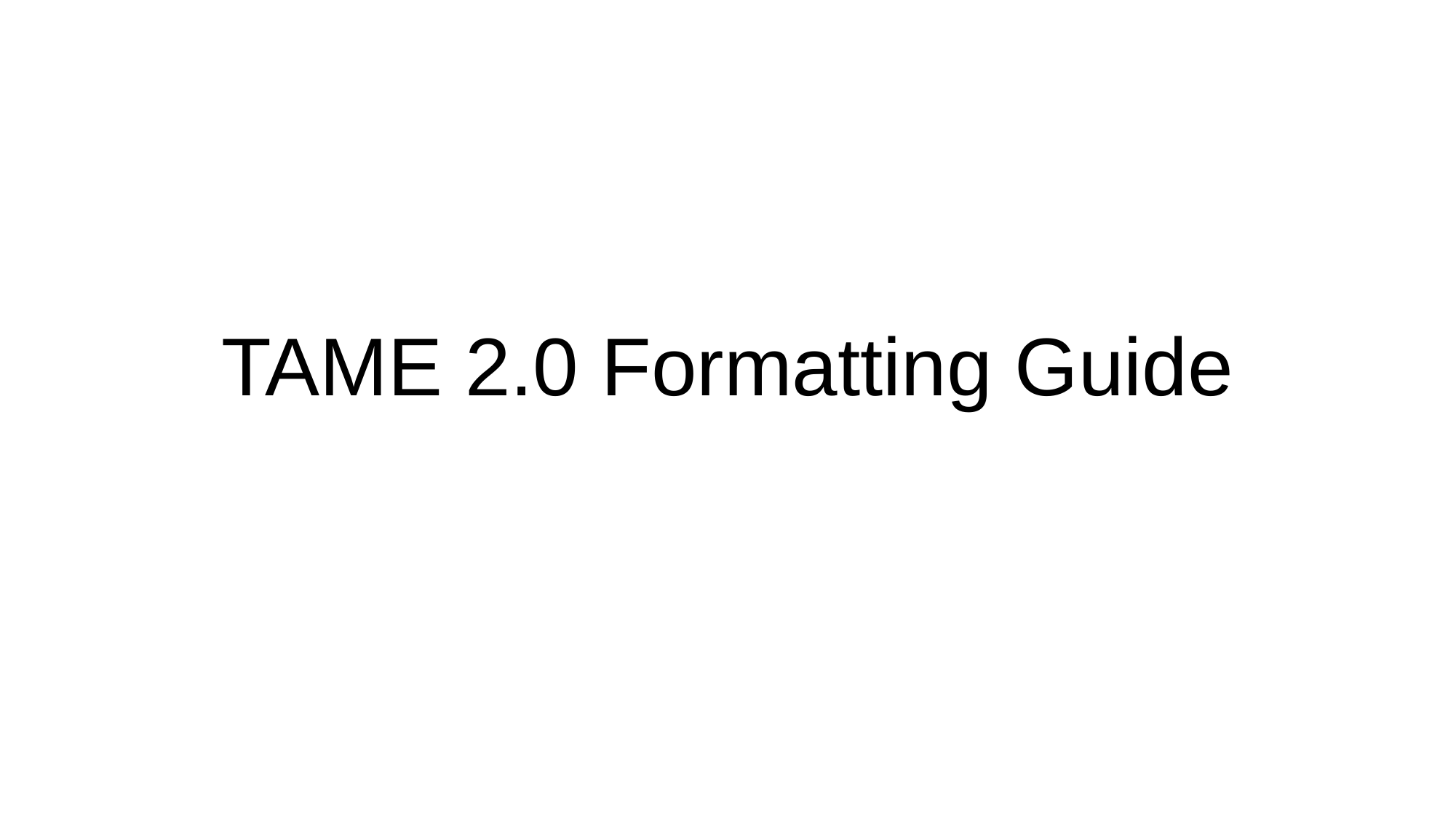

# TAME 2.0 Formatting Guide

## Slide 2
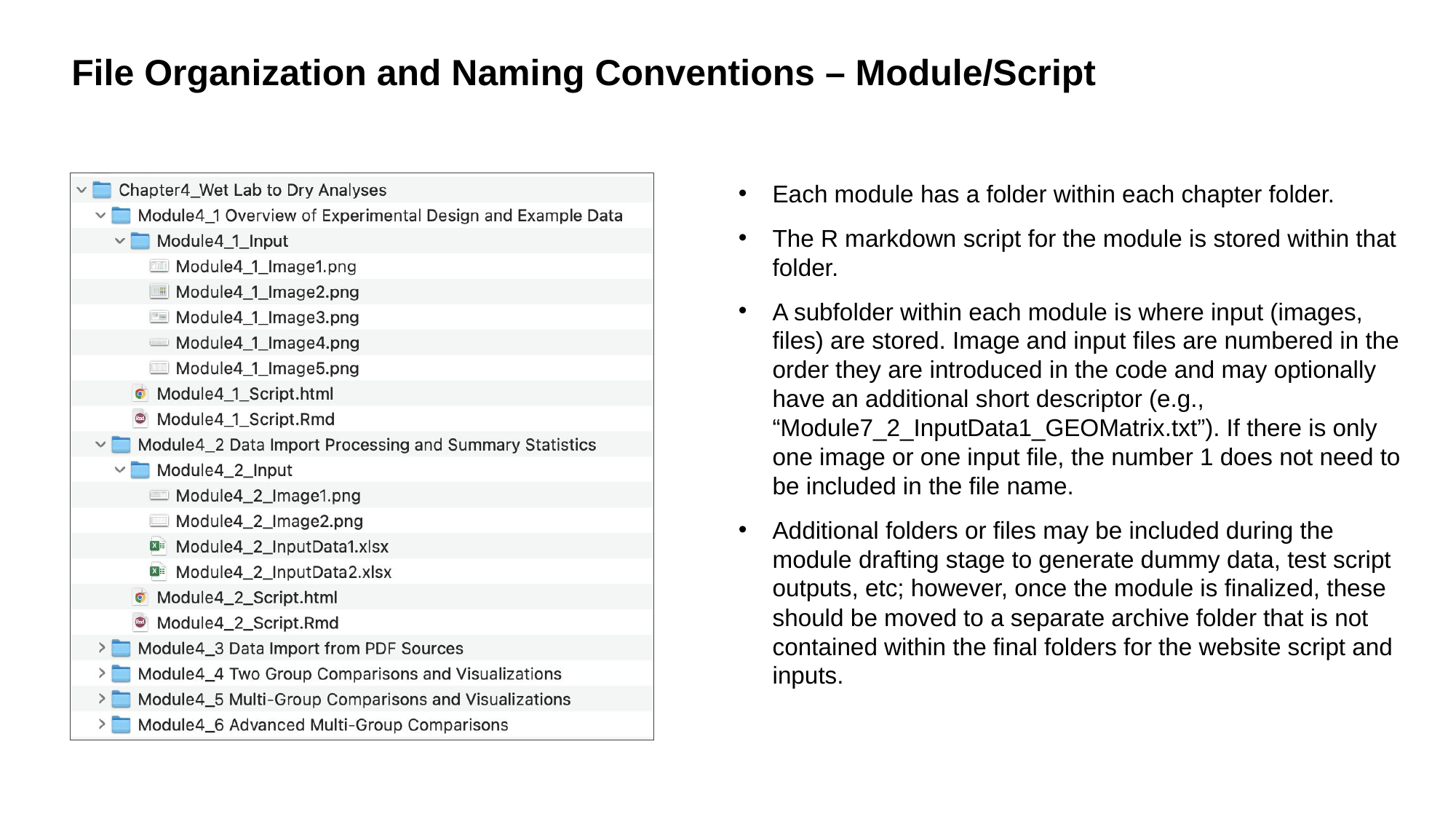

File Organization and Naming Conventions – Module/Script
Each module has a folder within each chapter folder.
The R markdown script for the module is stored within that folder.
A subfolder within each module is where input (images, files) are stored. Image and input files are numbered in the order they are introduced in the code and may optionally have an additional short descriptor (e.g., “Module7_2_InputData1_GEOMatrix.txt”). If there is only one image or one input file, the number 1 does not need to be included in the file name.
Additional folders or files may be included during the module drafting stage to generate dummy data, test script outputs, etc; however, once the module is finalized, these should be moved to a separate archive folder that is not contained within the final folders for the website script and inputs.

## Slide 3
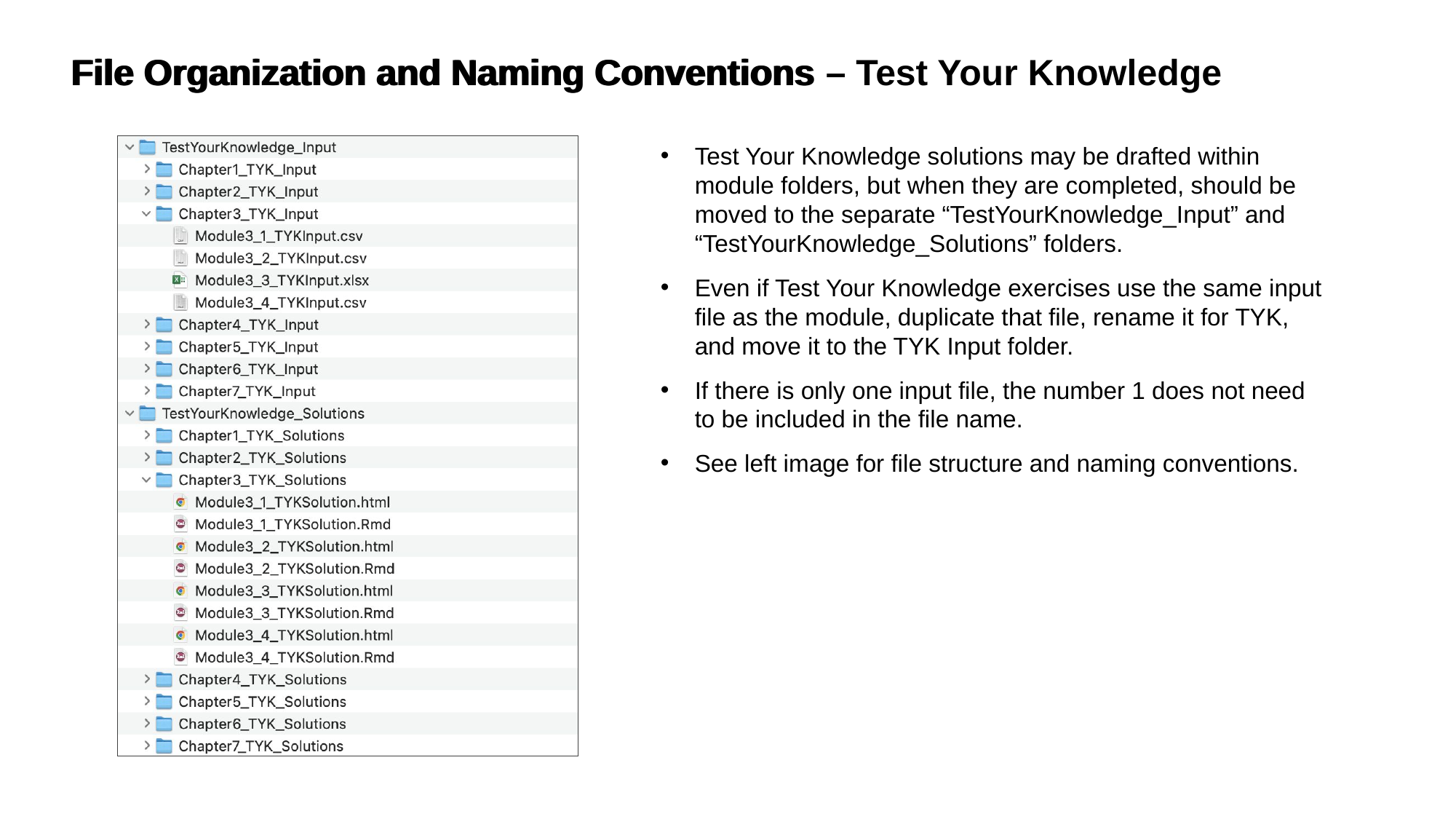

File Organization and Naming Conventions
File Organization and Naming Conventions – Test Your Knowledge
Test Your Knowledge solutions may be drafted within module folders, but when they are completed, should be moved to the separate “TestYourKnowledge_Input” and “TestYourKnowledge_Solutions” folders.
Even if Test Your Knowledge exercises use the same input file as the module, duplicate that file, rename it for TYK, and move it to the TYK Input folder.
If there is only one input file, the number 1 does not need to be included in the file name.
See left image for file structure and naming conventions.

## Slide 4
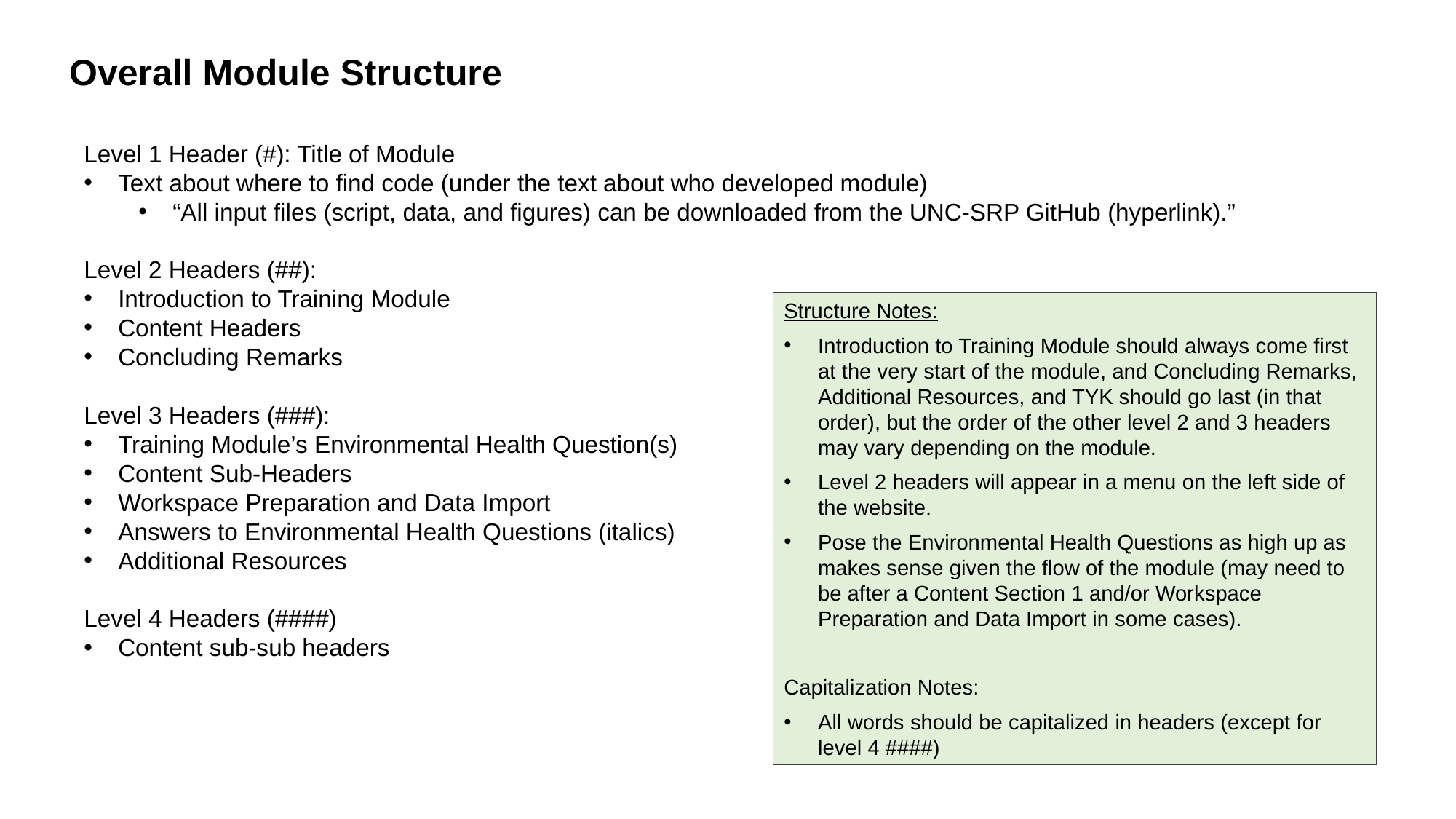

Overall Module Structure
Level 1 Header (#): Title of Module
Text about where to find code (under the text about who developed module)
“All input files (script, data, and figures) can be downloaded from the UNC-SRP GitHub (hyperlink).”
Level 2 Headers (##):
Introduction to Training Module
Content Headers
Concluding Remarks
Level 3 Headers (###):
Training Module’s Environmental Health Question(s)
Content Sub-Headers
Workspace Preparation and Data Import
Answers to Environmental Health Questions (italics)
Additional Resources
Level 4 Headers (####)
Content sub-sub headers
Structure Notes:
Introduction to Training Module should always come first at the very start of the module, and Concluding Remarks, Additional Resources, and TYK should go last (in that order), but the order of the other level 2 and 3 headers may vary depending on the module.
Level 2 headers will appear in a menu on the left side of the website.
Pose the Environmental Health Questions as high up as makes sense given the flow of the module (may need to be after a Content Section 1 and/or Workspace Preparation and Data Import in some cases).
Capitalization Notes:
All words should be capitalized in headers (except for level 4 ####)

## Slide 5
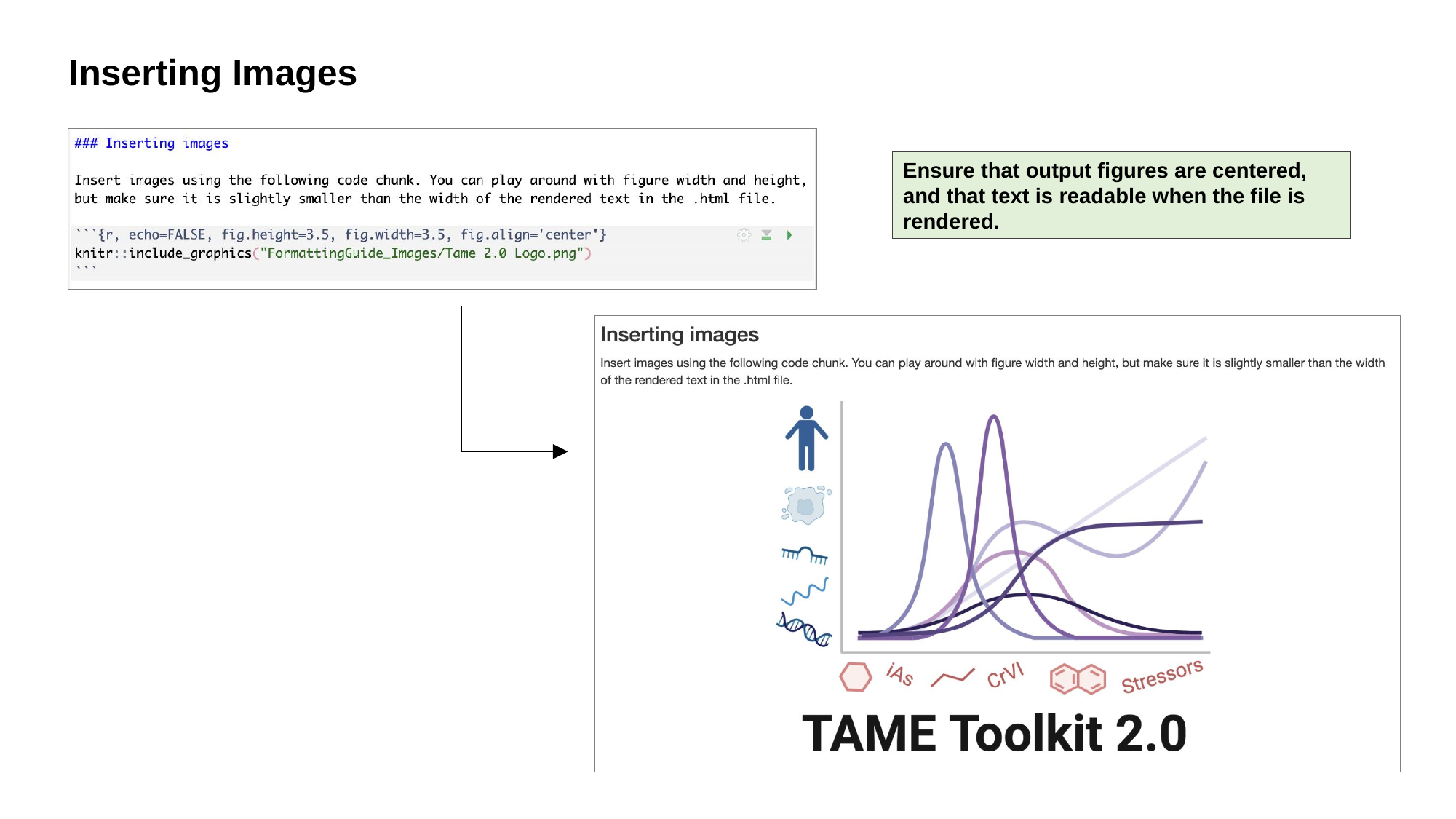

Inserting Images
Ensure that output figures are centered, and that text is readable when the file is rendered.

## Slide 6
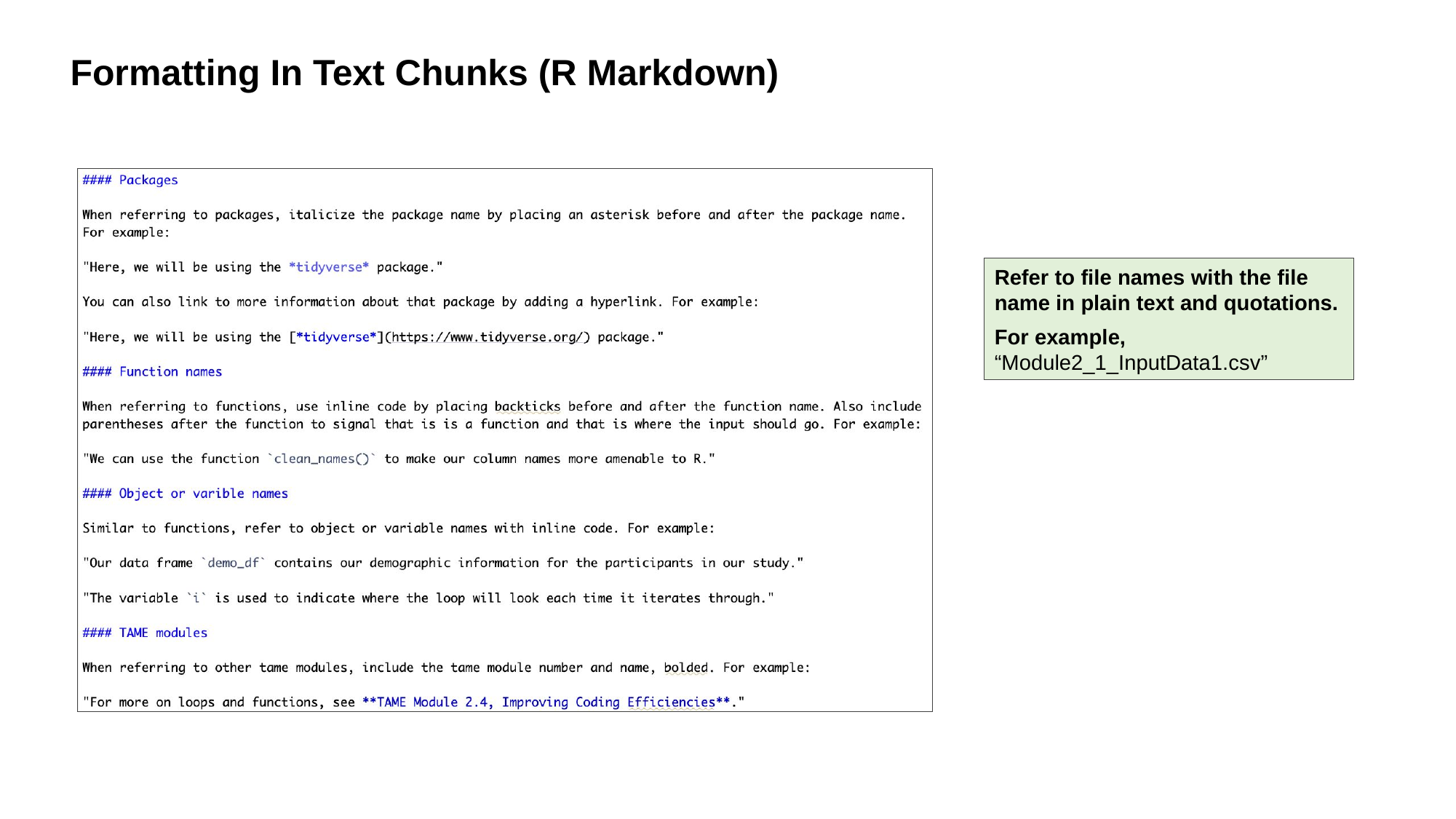

Formatting In Text Chunks (R Markdown)
Refer to file names with the file name in plain text and quotations.
For example, “Module2_1_InputData1.csv”

## Slide 7
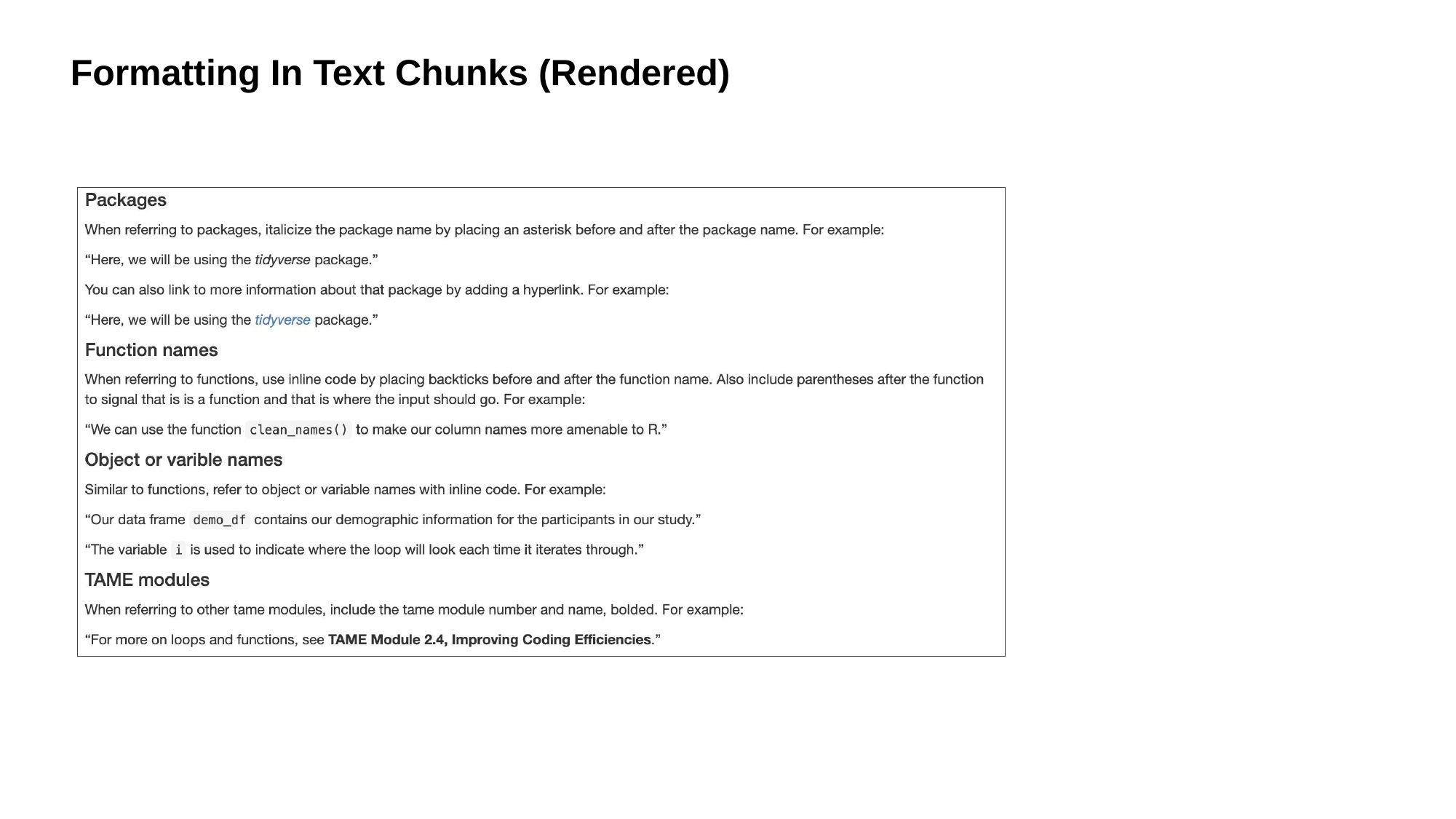

Formatting In Text Chunks (Rendered)

## Slide 8
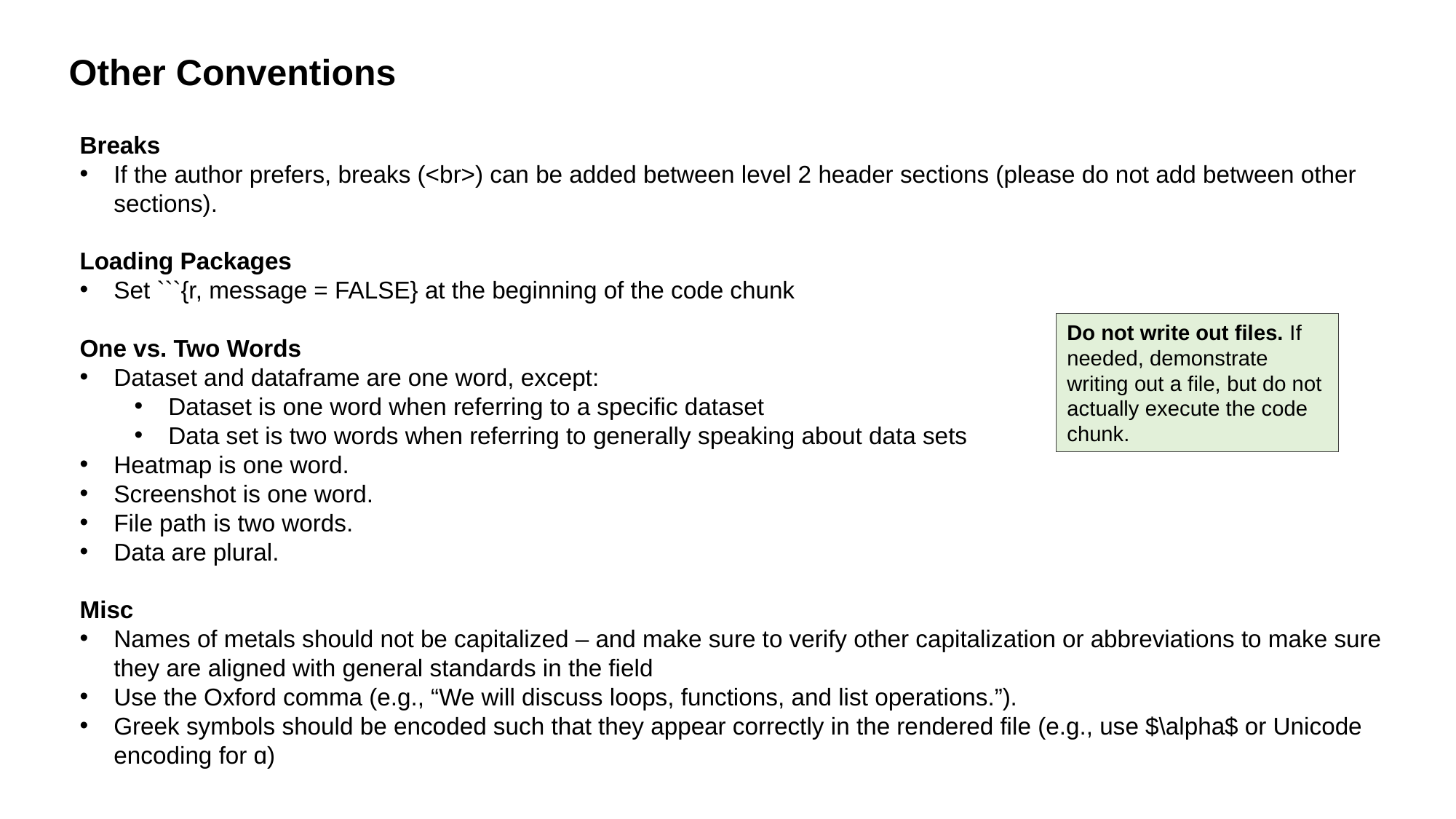

Other Conventions
Breaks
If the author prefers, breaks (<br>) can be added between level 2 header sections (please do not add between other sections).
Loading Packages
Set ```{r, message = FALSE} at the beginning of the code chunk
One vs. Two Words
Dataset and dataframe are one word, except:
Dataset is one word when referring to a specific dataset
Data set is two words when referring to generally speaking about data sets
Heatmap is one word.
Screenshot is one word.
File path is two words.
Data are plural.
Misc
Names of metals should not be capitalized – and make sure to verify other capitalization or abbreviations to make sure they are aligned with general standards in the field
Use the Oxford comma (e.g., “We will discuss loops, functions, and list operations.”).
Greek symbols should be encoded such that they appear correctly in the rendered file (e.g., use $\alpha$ or Unicode encoding for ɑ)
Do not write out files. If needed, demonstrate writing out a file, but do not actually execute the code chunk.

## Slide 9
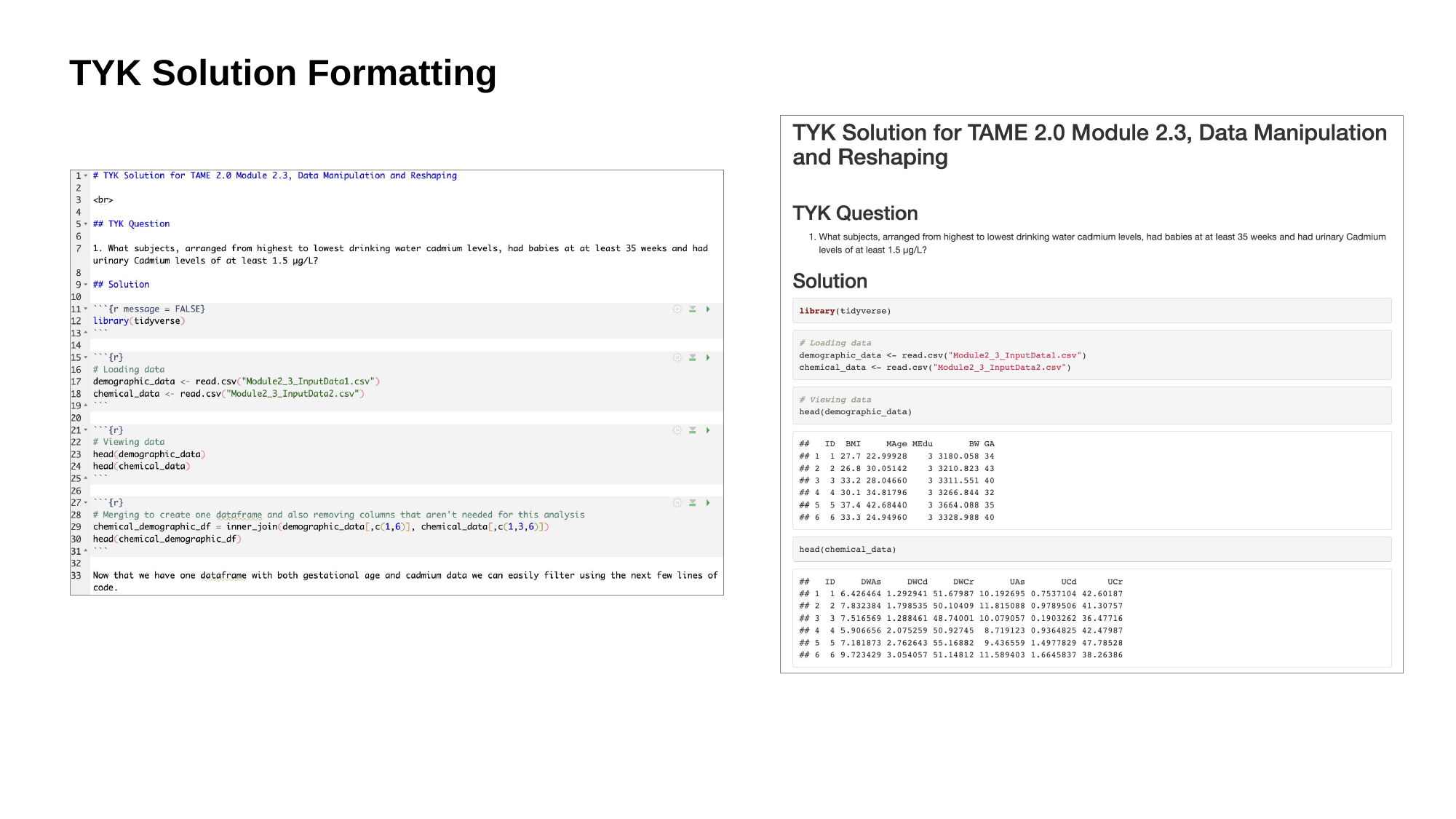

TYK Solution Formatting

## Slide 10
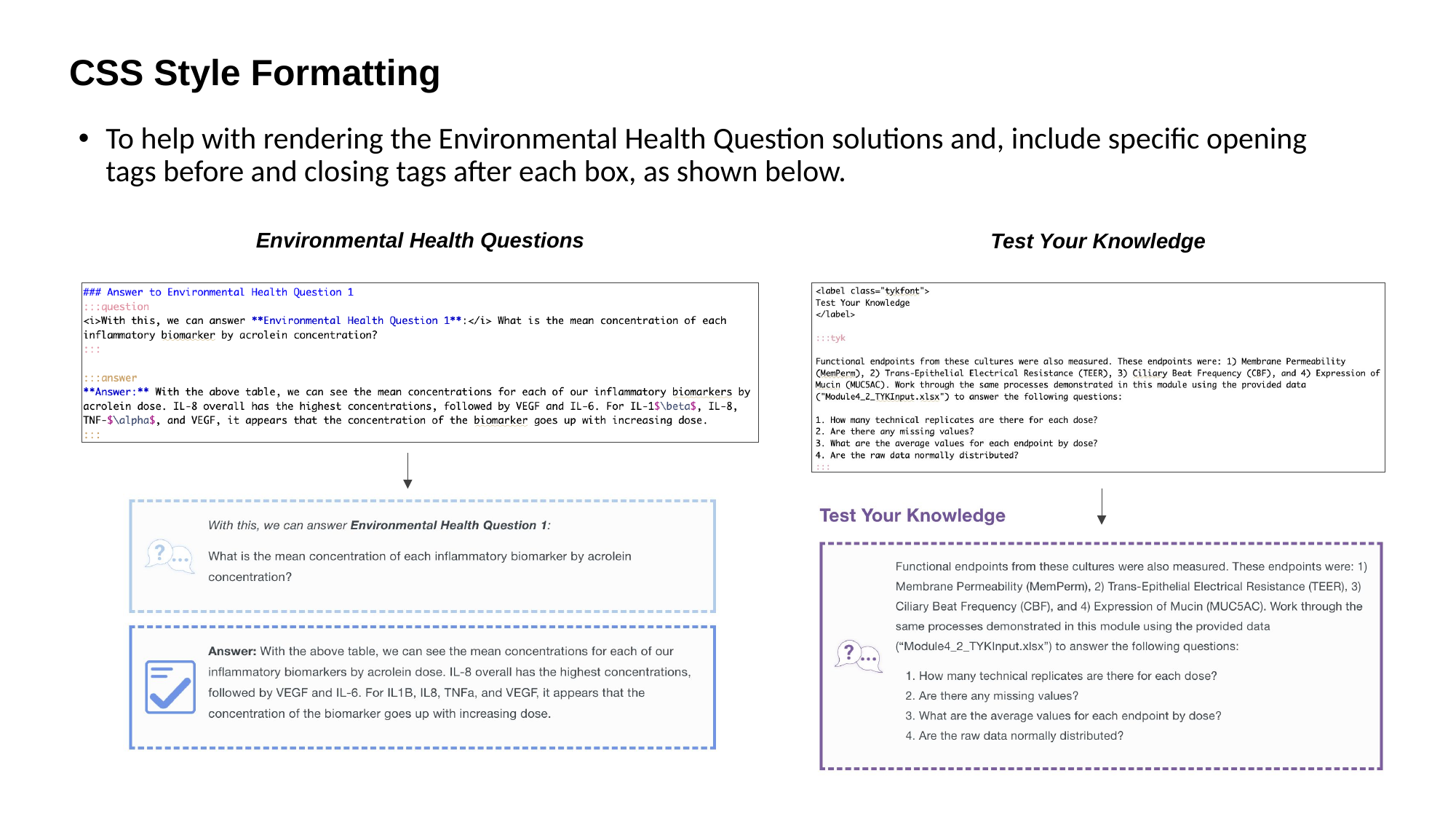

CSS Style Formatting
To help with rendering the Environmental Health Question solutions and, include specific opening tags before and closing tags after each box, as shown below.
Environmental Health Questions
Test Your Knowledge
