# Supplementary material for: TAME 2.0: expanding and improving online data science training for environmental health research
Source: Front Toxicol. 2025 Feb 12;7:1535098. doi: 10.3389/ftox.2025.1535098 (PMC11860945; doi:10.3389/ftox.2025.1535098)
Supplement: Supplementary file 3 [file DataSheet1.docx]

Supplementary Figures for “TAME 2.0: Expanding and Improving Online Data Science Training for Environmental Health Research”

|  |
| --- |
| **Figure S1. Changes in student pre- and post-course survey results from UNC ‘ENVR730: Computational Toxicology and Exposure Science’.** Students filled out a survey before beginning the course and after completing the course to understand changes in their knowledge relating to computational approaches in toxicology and exposure science. Statistical significance was determined using paired Wilcox tests. N = 25 students with matching pre/post results. ** p < 0.01, *** p < 0.001, **** p < 0.0001. |

| 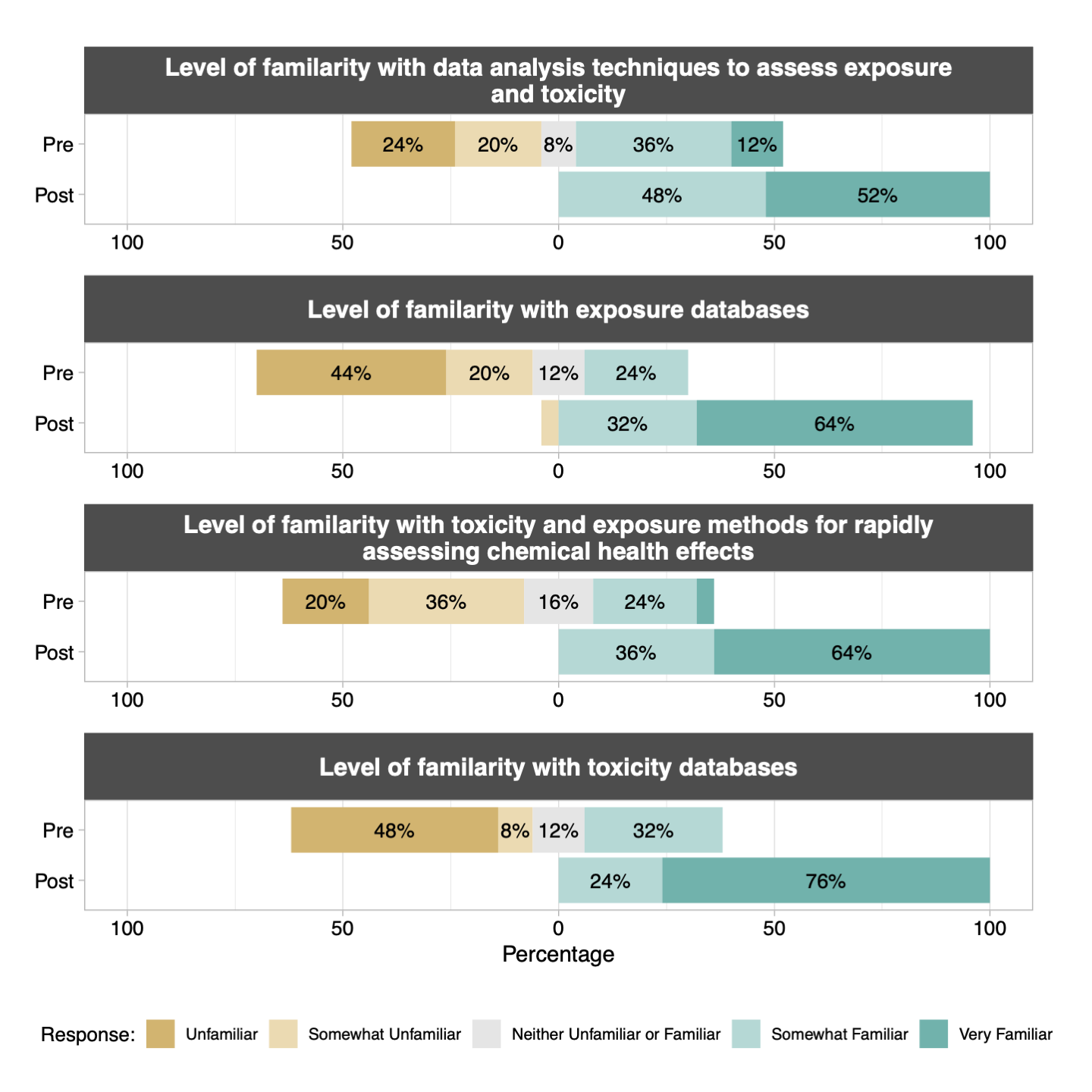 |
| --- |
| **Figure S2. Pre- and post-course student survey results from UNC ‘ENVR730: Computational Toxicology and Exposure Science’: Full results for Question Set 1**. Students filled out a survey before beginning the course and after completing the course to understand changes in their knowledge. N = 25 students with matched pre/post results. |

| 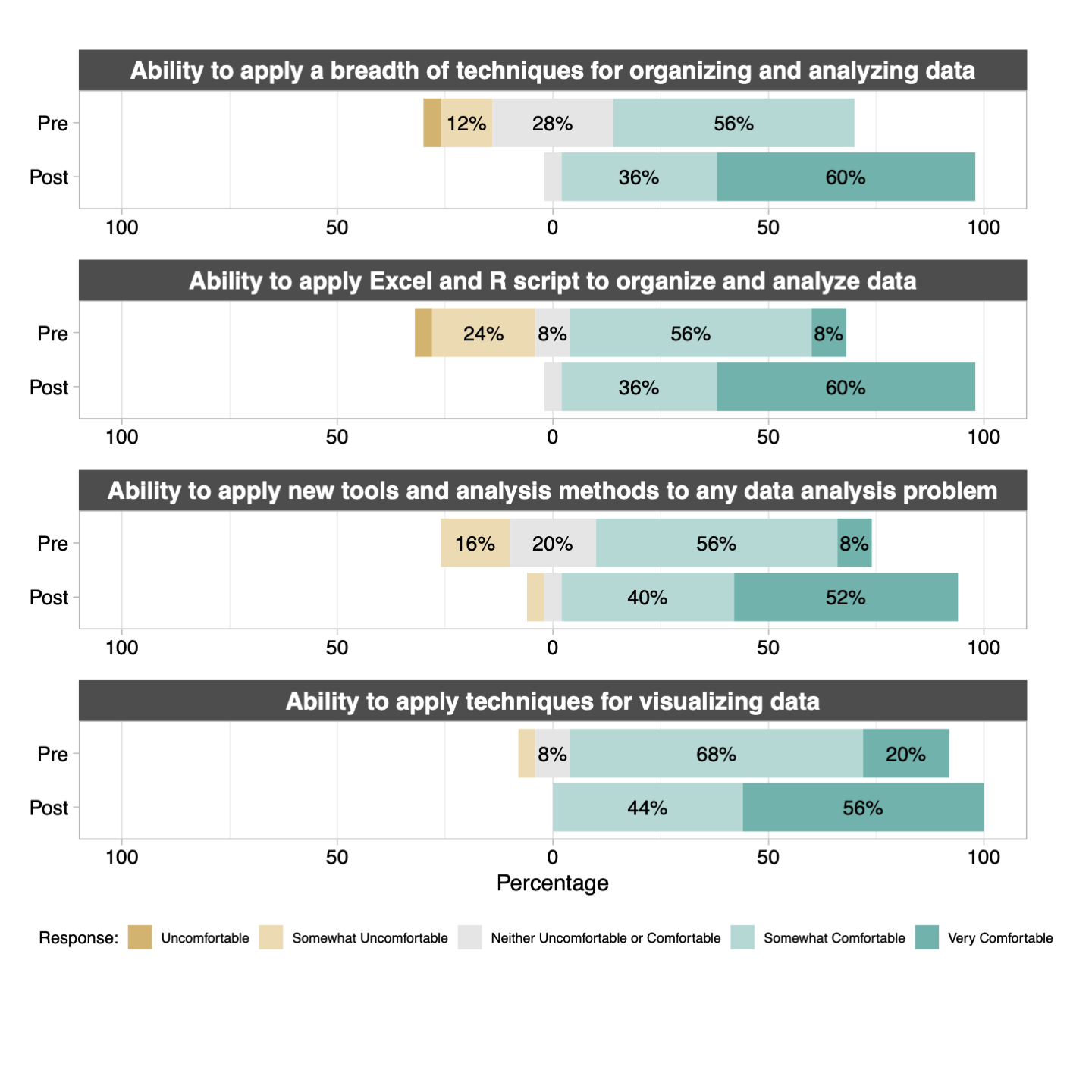 |
| --- |
| **Figure S3. Pre- and post-course student survey results from UNC ‘ENVR730: Computational Toxicology and Exposure Science’: Full results for Question Set 2**. Students filled out a survey before beginning the course and after completing the course to understand changes in their knowledge. N = 25 students with matched pre/post results. |

| 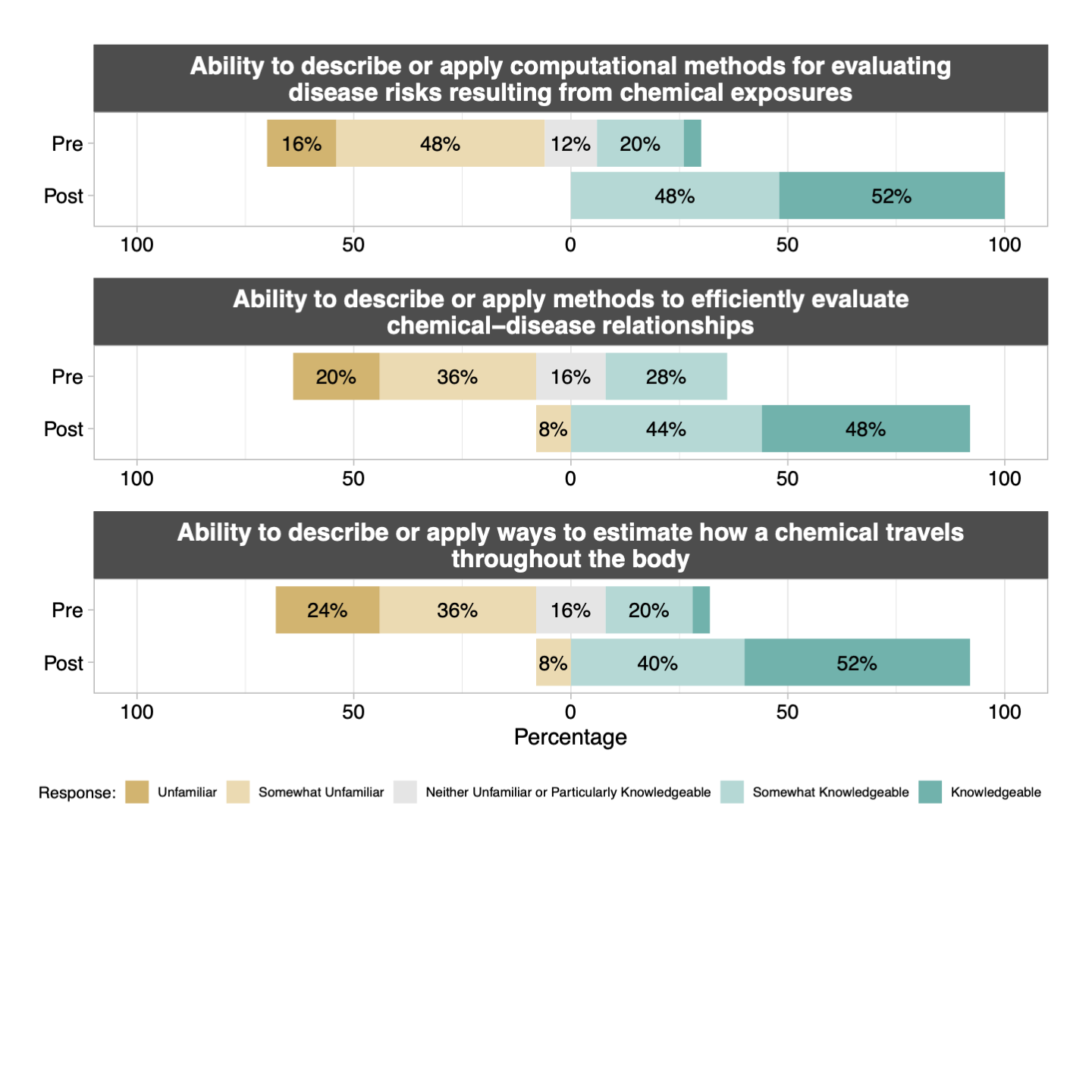 |
| --- |
| **Figure S4. Pre- and post-course student survey results from UNC ‘ENVR730: Computational Toxicology and Exposure Science’: Full results for Question Set 3**. Students filled out a survey before beginning the course and after completing the course to understand changes in their knowledge. N = 25 students with matched pre/post results. |
